# A human-specific regulatory mechanism revealed in a pre-implantation model

**DOI:** 10.1038/s41586-025-09571-1

**Published:** 2025-10-01

**Authors:** Raquel Fueyo, Sicong Wang, Olivia J. Crocker, Tomek Swigut, Hiromitsu Nakauchi, Joanna Wysocka

**Affiliations:** 1 Department of Chemical and Systems Biology, Stanford University School of Medicine, Stanford, CA USA; 2 Institute of Stem Cell Biology and Regenerative Medicine, Stanford University School of Medicine, Stanford, CA USA; 3 Department of Genetics, Stanford University School of Medicine, Stanford, CA USA; 4 Stem Cell Therapy Laboratory, Advanced Research Institute, Tokyo Medical and Dental University, Tokyo, Japan; 5 Department of Developmental Biology, Stanford University School of Medicine, Stanford, CA USA; 6 Howard Hughes Medical Institute, Stanford University School of Medicine, Stanford, CA USA

**Keywords:** Gene regulation, Stem cells

## Abstract

Stem cell-based human embryo models offer a unique opportunity for functional studies of the human-specific features of development. Here we genetically and epigenetically manipulate human blastoids, a 3D embryo model of the blastocyst^1^, to investigate the functional effect of HERVK LTR5Hs, a hominoid-specific endogenous retrovirus, on pre-implantation development. We uncover a pervasive *cis*-regulatory contribution of LTR5Hs elements to the hominoid-specific diversification of the epiblast transcriptome in blastoids. Many of the LTR5Hs genomic insertions in the human genome are unique to our own species. We show that at least one such human-specific LTR5Hs element is essential for the blastoid-forming potential via enhancing expression of the primate-specific *ZNF729* gene, encoding a KRAB zinc-finger protein. ZNF729 binds to GC-rich sequences, abundant at gene promoters associated with basic cellular functions, such as cell proliferation and metabolism. Despite mediating recruitment of TRIM28, at many of these promoters ZNF729 acts as a transcriptional activator. Together, our results illustrate how recently emerged transposable elements and genes can confer developmentally essential functions in humans.

## Abstract (web summary)

Genetic manipulation of blastoids reveals the role of recently emerged transposable elements and genes in human development.

## Main

**Extended Data Fig. 1:**
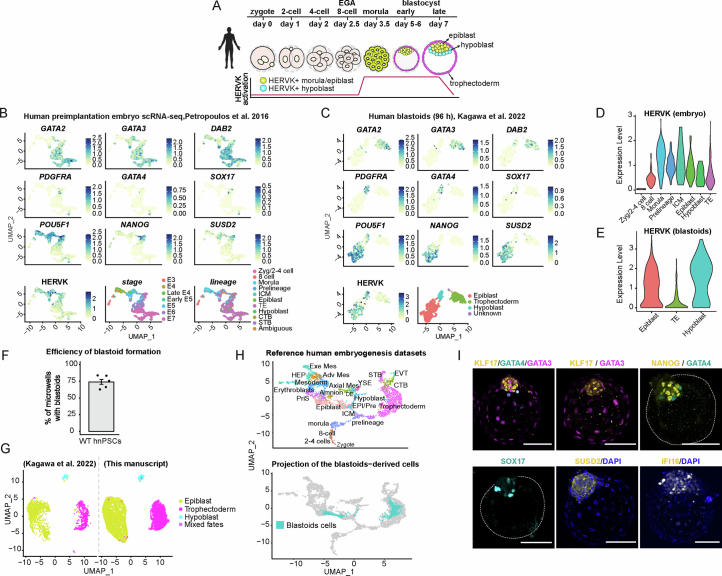
Benchmarking a human blastoid protocol to study HERVK in human pre-implantation development. **a**. Stages of human pre-implantation development. HERVK expression levels from^9,18^ are represented by a red line. HERVK is active in the epiblast (yellow cells) and the hypoblast (cyan cells). EGA: embryonic genome activation. The silhouette of the human was created in BioRender. Fueyo, R. (2025) https://BioRender.com/tmgd0pn. **b**. UMAP of expression of the indicated lineage markers and HERVK in human pre-implantation single cells. Bottom right two panels display annotation of data using stage (the prefix E is equivalent to days) or lineages (data and annotations from refs. ^18,51^). **c**. UMAP of expression of the indicated lineage markers and HERVK in single cells dissociated from blastoids at 96 h. Bottom right panels display annotation of data using lineages (data and annotations from ref. ^17^). **d**. and **e**. Violin plots of HERVK normalized expression levels at the different lineages present in human pre-implantation embryos^18^ (**d**) or 96 h blastoids^17^ (**e**). **f**. Bar plot displays efficiency of blastoid formation with wild-type hnPSCs, n = 5 biologically independent replicates. Data represents mean values +/− SEM. **g**. UMAPs representing integrated single cell RNA-seq datasets from the previously reported human blastoid derivation protocol (Kagawa et al.^17^) and blastoids generated and benchmarked in this manuscript. Colors represent the blastoid lineages. **h**. UMAP of a reference collection of human embryo single cell RNA-seq transcriptomes^51^ (top) and projection of cells from blastoids derived in this manuscript (bottom). **i**. Representative confocal images (n ≥ 3) of immunostaining of blastoids derived from wild-type hnPSCs with lineage-specific markers: KLF17, NANOG, SUSD2, IFI16 (yellow, epiblast), GATA4, SOX17 (cyan, hypoblast), and GATA3 (magenta, trophectoderm). White bar represents 100 µm. Source Data

Endogenous retroviruses (ERVs), also called long terminal repeat (LTR) retrotransposons, comprise approximately 8.9% of the human genome^2^. ERVs are remnants of ancient retroviral infections of the germline that integrated into the genome, propagated vertically and ultimately became fixed in the population^3^. To successfully endogenize, the LTRs of ERVs must have entered the host genome able to engage the transcriptional machinery in germ cells or pre-implantation embryo cells. This, and the reduced DNA methylation, can potentially account for the widespread regulatory co-option of ERVs in mammalian pre-implantation development^4^, a period that spans the time from fertilization to the attachment of the blastocyst to the uterus. For example, during mouse pre-implantation, many LTRs function as stage-specific promoters^5–8^. In humans, ERVs of the HERVK (HML-2) family—specifically those of the LTR5Hs subtype—are transcriptionally activated in the embryo around the eight-cell stage and stay active in the blastocyst^9^ (Extended Data Fig. 1a). HERVK LTR5Hs is also active in human teratocarcinoma and human naive pluripotent stem cells (hnPSCs) where it exerts enhancer function^10,11^. HERVK LTR5Hs is the evolutionarily most recent ERV in humans. It first invaded the genome after the split of hominoids (apes) from Old World monkeys, and it remained active following the split of humans and chimpanzees^12^. As a result, the approximately 700 LTR5Hs insertions in the human genome are unique to hominoids, with a subset being specific to humans^12,13^. Nonetheless, the functional effect of HERVK LTR5Hs on pre-implantation development remains poorly understood.

Although the general principles of early development are conserved across mammals, many aspects have diverged between species^14,15^. Ethical and practical considerations limit functional studies in human embryos^16^. However, groundbreaking work established stem cell-based 3D embryo models named blastoids, which recapitulate the morphology and lineage specification of the human blastocyst (reviewed in ref. ^1^). Although not without limitations, human blastoids offer unprecedented opportunities to study species-specific features of human pre-implantation. Here we perturbed the function of HERVK LTR5Hs in human blastoids and reveal its dose-dependent effect on blastoid formation and gene regulation. We further uncovered a human-specific LTR5Hs insertion that enhances expression of the gene encoding the zinc-finger transcription factor ZNF729, promotes proliferation of hnPSCs and is essential for endowing hnPSCs with blastoid-forming potential. We demonstrate that ZNF729 binds to and regulates GC-rich promoters of genes involved in fundamental cellular functions. Together, our work reveals an evolutionary novel mechanism regulating conserved cellular processes and paves the way for systematic interrogation of transposable element function using in vitro human embryo models.

## HERVK LTR5Hs impacts blastoid formation

**Fig. 1:**
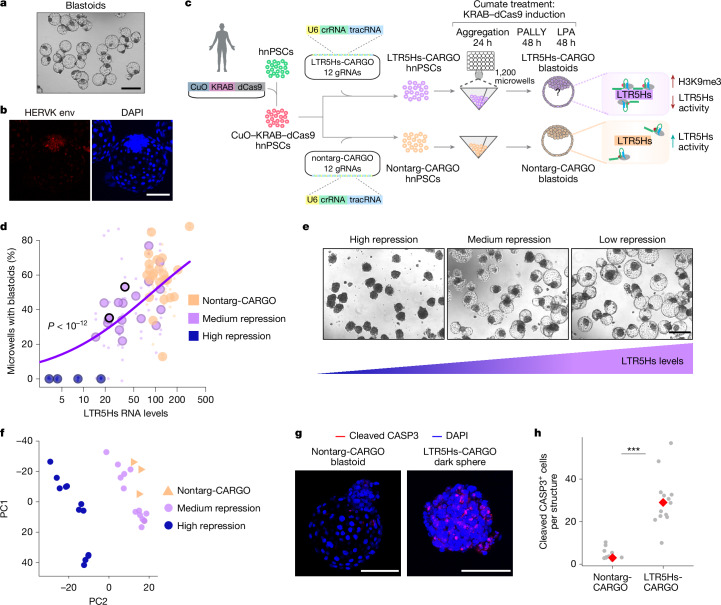
LTR5Hs activity contributes to the blastoid formation potential of hnPSCs. **a**, Representative bright-field image of wild-type blastoids (*n* = 3 biological replicates). Scale bar, 400 µm. **b**, Representative confocal images of a wild-type blastoid immunostained with HERVK envelope (env) antibodies and DAPI (*n* = 4 biological replicates). Scale bar, 100 µm. **c**, Schematic of nontarg-CARGO and LTR5Hs-CARGO hnPSCs and the generation of blastoids. The cartoon summarizes how LTR5Hs-CARGO promotes H3K9me3 deposition and LTR5Hs repression (right). crRNA, CRISPR RNA; LPA, media containing 1-oleoyl lysophosphatidic acid sodium salt; PALLY, media containing PD0325901, A83-01, leukemia inhibitory factor, LPA and Y-27632; tracRNA, *trans*-activating crRNA. The silhouette of the human was created in BioRender. Fueyo, R. (2025) https://BioRender.com/tmgd0pn. **d**, Blastoid-forming potential of hnPSC depends on LTR5Hs activity (β-regression, two-sided log-likelihood test = 2.39 × 10^−13^). Blastoid formation was assessed in 23 LTR5Hs-CARGO (*n* = 3 biological replicates) and 24 nontarg-CARGO (*n* = 2 biological replicates) clonal cell lines. Plotted is the proportion of blastoids per well versus Taqman LTR5Hs RNA levels normalized to *RPL13A* RNA levels. The purple line represents the LTR5Hs-CARGO regression line. The purple circles with black outlines indicate clones used for scRNA-seq experiments. **e**, Bright-field images of structures collected upon LTR5Hs high repression (left; dark spheres), medium repression (middle; dark spheres/blastoid-like structures) and no LTR5Hs repression (right; blastoid-like structures). Images are representative of 23 LTR5Hs-CARGO (*n* = 3 biological replicates) and 24 nontarg-CARGO (*n* = 2 biological replicates) clonal cell lines. Scale bar, 400 µm. **f**, PCA of the bulk RNA-seq transcriptomes obtained from nontarg-CARGO and LTR5Hs-CARGO clonal cell lines with high or medium expression levels (total of 11 clonal cell lines in biological duplicates or triplicates). **g**, Representative images of blastoid (left; *n* = 11 from three biological replicates) or dark sphere (right; *n* = 13 from three biological replicates) immunostained with cleaved CASP3 (red) and DAPI (blue). Scale bars, 100 µm. **h**, Quantification of cleaved CASP3 immunostainings described in panel **g**. Red diamonds display the median values. Significance was determined by an unpaired, two-tailed Student’s *t*-test: *P* = 8.5 × 10^−^^7^. Source Data

To probe the phenotypic effect of HERVK LTR5Hs activity on human pre-implantation, we turned to a human blastoid model^17^ (Fig. 1a). Blastoids contain analogues to the blastocyst lineages: the epiblast that gives rise to the embryo proper, the trophectoderm that generates the placenta, and the hypoblast that develops into the yolk sac^14^. Analysis of the HERVK expression pattern in human embryos and blastoids using published single-cell RNA sequencing (scRNA-seq) datasets^17,18^ revealed high HERVK expression in the epiblast and hypoblast lineages and low expression in the trophectoderm (Extended Data Fig. 1b–e).

We implemented the blastoid generation protocol^17^ from hnPSCs, obtaining approximately 70% efficiency (Fig. 1a, Extended Data Fig. 1f and Supplementary Video 1). scRNA-seq analysis of the generated blastoids revealed transcriptome profiles consistent with the presence of the three blastocyst lineages and absence of the post-implantation embryo lineages (Extended Data Fig. 1g,h). We also performed immunostainings with markers of the epiblast (KLF17, NANOG, SUSD2 and IFI16)^18–20^; the trophectoderm (GATA3)^18,21^; the hypoblast (SOX17 and GATA4)^18,22^; and the HERVK envelope (Fig. 1b, Extended Data Fig. 1i and Supplementary Table 1), all of which showed staining patterns consistent with human blastocysts^9,19,21,22^.

**Extended Data Fig. 2:**
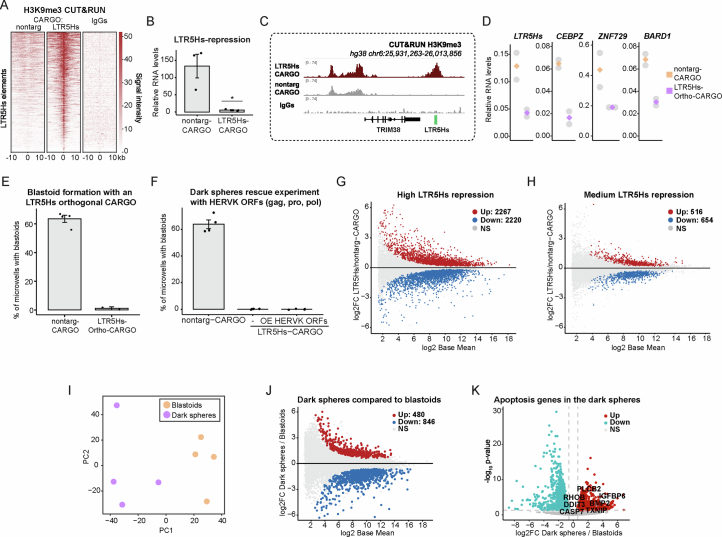
LTR5Hs-driven gene regulatory changes underlie blastoid formation potential. **a**. Heatmaps displaying H3K9me3 CUT&RUN signal over LTR5Hs elements in nontarg-, LTR5Hs-CARGO cells, and in the IgGs negative control. **b**. RT-qPCR depicting LTR5Hs RNA levels measured with Taqman and normalized to the *RPL13* gene in nontarg-CARGO and LTR5Hs-CARGO cells, n = 3, unpaired two-tailed Student’s t-test, *P* = 0.02. Data represents mean values +/− SEM. **c**. IGV genome browser capture of H3K9me3 CUT&RUN profiles over an LTR5Hs insertion. **d**. Plots depict qPCR RNA levels of LTR5Hs, and LTR5Hs-regulated genes in nontarg-CARGO and LTR5Hs-Ortho-CARGO hnPSCs, (n = 2). **e**. Blastoid formation efficiencies of nontarg-CARGO and LTR5Hs-Ortho-CARGO hnPSCs (n = 4 biological replicates for nontarg-CARGO and n = 2 for LTR5Hs-Ortho CARGO). Data represents mean values +/− SEM. **f**. Blastoid formation efficiencies of two nontarg-CARGO hnPSCs clones, two LTR5Hs-CARGO high repression clones, and two LTR5Hs-CARGO high repression clones expressing a transgene encoding the HERVK proteins gag, pro, and pol (n = 2 biological replicates). Data represents mean values +/− SEM. **g**. MA plot representing bulk RNA-seq results of LTR5Hs-CARGO high repression clones (n = 4 clones, in biological triplicates) compared to nontarg-CARGO clones (n = 3). FDR 5%. **h**. MA plot representing bulk RNA-seq results of LTR5Hs-CARGO medium repression clones (n = 5, in biological duplicates or triplicates) compared to nontarg-CARGO clones. FDR 5%. **i**. PCA of the bulk RNA-seq transcriptomes obtained from blastoids and dark spheres upon LTR5Hs-CARGO induction for 96 h (n = 4). **j**. MA plot representing bulk RNA-seq results of blastoids and dark spheres obtained upon LTR5Hs repression (n = 4) FDR 5%. **k**. Volcano plot of bulk RNA-seq of dark spheres compared to blastoids (n = 4). Labeled dots represent statistically significant genes involved in apoptosis according to GSEA curated gene sets^25^. DESeq2 Wald test FDR 5%. P-values are corrected for multiple testing using the Benjamini and Hochberg method^52^. Source Data

We previously reported that CARGO-CRISPRi allows for efficient and selective perturbation of HERVK LTR5Hs function across the genome^10,23^. In brief, a 12-mer guide RNA (gRNA) array was designed to target the majority of 697 LTR5Hs instances (referred to as LTR5Hs-CARGO), along with a control non-targeting array (nontarg-CARGO). To study the functional effect of HERVK LTR5Hs repression on human blastoid formation, we generated hnPSCs expressing a cumate-inducible catalytically dead version of Cas9 (dCas9) fused to the transcriptional repressor KRAB (KRAB–dCas9) and then introduced LTR5Hs-CARGO or nontarg-CARGO arrays to generate clonal cell lines (Fig. 1c). We confirmed that the induction of KRAB–dCas9 in LTR5Hs-CARGO hnPSCs but not in nontarg-CARGO hnPSCs resulted in H3K9me3 deposition across the majority of LTR5Hs instances and in the repression of LTR5Hs-originating transcripts (Extended Data Fig. 2a–c).

We next asked how LTR5Hs repression affects blastoid formation by inducing blastoid generation from 24 and 23 distinct nontarg-CARGO and LTR5Hs-CARGO clonal cell lines, respectively (Fig. 1c). We then measured blastoid formation efficiency as a function of LTR5Hs expression levels utilizing TaqMan probes (Fig. 1c–e and Supplementary Table 1). We observed a correlation between LTR5Hs expression and blastoid-forming potential, with high repression of LTR5Hs activity being incompatible with blastoid formation and instead resulting in structures resembling dark spheres. At intermediate levels of repression, blastoid-like structures still formed, albeit at reduced efficiencies, whereas lines with poor LTR5Hs repression formed blastoids at efficiencies comparable with the nontarg-CARGO hnPSCs. To rule out off-target effects, we cloned a new gRNA array, orthogonal to the LTR5Hs-CARGO array (named LTR5Hs-Ortho-CARGO) capable of repressing LTR5Hs and its selected gene targets (Extended Data Fig. 2d). In agreement with the LTR5Hs-CARGO, LTR5Hs-Ortho-CARGO hnPSCs also failed to form blastoids (Extended Data Fig. 2e).

HERVK retains coding capacity for viral proteins that can be detected in human embryos^9^. To explore whether loss of these viral proteins is responsible for the blastoid formation failure, we performed rescue experiments by genomic integration of an active transgene encoding the HERVK viral proteins gag, pro and pol^24^. This transgene failed to restore the blastoid formation capacity of high LTR5Hs repression hnPSCs, suggesting that HERVK viral proteins alone are not responsible for the dark spheres phenotype (Extended Data Fig. 2f). Together, these results indicate that LTR5Hs activity impacts blastoid formation and suggest a non-neutral contribution of HERVK to human pre-implantation.

## Gene misregulation upon LTR5Hs repression

The different blastoid formation capacity of high and medium LTR5Hs repression hnPSCs prompted us to analyse their underlying gene expression changes by RNA-seq after 96 h of repression (we note that hnPSCs cannot be maintained long term after induction of LTR5Hs repression). Principal component analysis (PCA) revealed that although all LTR5Hs-CARGO clones separated from the nontarg-CARGO clones, the medium repression clones clustered much closer to the controls than the high repression clones (Fig. 1f). Indeed, we confirmed a stronger misregulation of gene expression in high versus medium repression clones than nontarg-CARGO controls, in both the number of affected genes and the magnitude of the responses (Extended Data Fig. 2g,h). Gene Ontology of transcripts dysregulated in the high but not in the medium repression clones revealed categories of embryo morphogenesis and immune response among others, suggesting that the high-repressing clones undergo additional gene dysregulation (Supplementary Table 2).

The dark spheres obtained upon near-full LTR5Hs repression do not cavitate and look homogeneous under bright field (Fig. 1e). Bulk RNA-seq of the dark spheres compared with bulk RNA-seq of control blastoids detected a clear separation of these transcriptomes in the PCA space and identified differentially expressed genes (Extended Data Fig. 2i,j). Gene Ontology analysis of these transcripts revealed categories related to morphogenesis, migration, cell proliferation and others (Supplementary Table 2). Among the upregulated genes, some transcripts pointed to apoptosis (for example, *CASP7*), thus we systematically investigated whether apoptotic genes were differentially regulated. Indeed, comparison with a set of curated apoptosis genes^25^ identified upregulation of apoptotic gene transcripts in the dark spheres (Extended Data Fig. 2k). To confirm this, we stained blastoids and dark spheres with the apoptotic marker cleaved CASP3. Although each blastoid displayed a median of three cleaved CASP3^+^ cells, for dark spheres, the median was 29 (Fig. 1g,h). This demonstrates that under high LTR5Hs repression, hnPSCs undergo widespread gene expression changes incompatible with blastoid formation and consistent with an apoptotic phenotype.

## LTR5Hs repression affects lineage identity

**Fig. 2:**
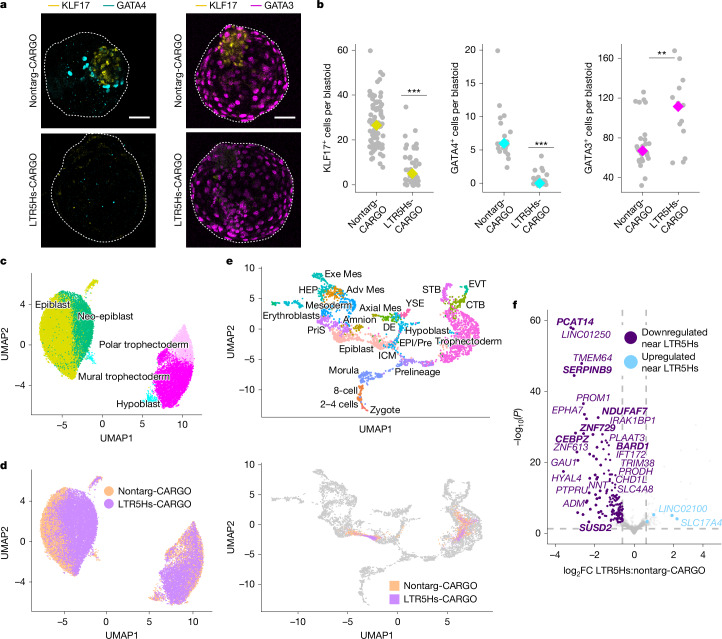
LTR5Hs activity is required for proper lineage acquisition in blastoids. **a**, Representative confocal images of nontarg-CARGO and LTR5Hs-CARGO blastoids stained with lineage-specific antibodies (KLF17 (an epiblast marker) is in yellow, *n* = 78 for nontarg-CARGO and *n* = 41 for LTR5Hs-CARGO; GATA4 (a hypoblast marker) is in cyan, *n* = 22 for nontarg-CARGO and *n* = 24 for LTR5Hs-CARGO; GATA3 (a trophectoderm marker) is in magenta, *n* = 27 for nontarg-CARGO and *n* = 14 for LTR5Hs-CARGO). Stained blastoids represent *n* ≥ 4 biological replicates. Scale bars, 50 µm. **b**, Counting of cells showing positive staining for the indicated markers in the nontarg-CARGO and LTR5Hs-CARGO blastoids as detailed in panel **a**. The grey dots represent the number of cells in individual blastoids. The yellow, cyan or magenta diamonds represent the median. Counted blastoids were generated in *n* ≥ 4 biological replicates; specific numbers counted are as follows: KLF17, *n* = 78 for nontarg-CARGO and *n* = 41 for LTR5Hs-CARGO; GATA4, *n* = 22 for nontarg-CARGO and *n* = 24 for LTR5Hs-CARGO; and GATA3, *n* = 27 for nontarg-CARGO and *n* = 14 for LTR5Hs-CARGO. Significance was determined using an unpaired, two-tailed Student’s *t*-test for KLF17 (*P* = 2.2 × 10^−16^), GATA4 (*P* = 3.69 × 10^−10^) and GATA3 (*P* = 0.001). **c**,**d**, Uniform manifold and approximation projection (UMAP) of transcriptomes from single cells dissociated from nontarg-CARGO and LTR5Hs-CARGO blastoids. The colours indicate cells belonging to the same lineage-specific clusters, as specified (**c**), or indicate the genotype of origin (orange for nontarg-CARGO blastoids and purple for LTR5Hs-CARGO blastoids; **d**). **e**, UMAP of a reference collection of human embryo scRNA-seq transcriptomes^51^ (top) and projection of nontarg-CARGO (orange) or LTR5Hs-CARGO (purple) PIP-seq results in that UMAP (bottom). Adv Med, advanced mesoderm; Axial Mes, axial mesoderm; CTB, cytotrophoblast; DE, definitive endoderm; EPI/Pre, epiblast prelineage; EVT, extravillous trophoblast; Exe Mes, extraembryonic mesoderm; HEP, haemato-endothelial progenitors; ICM, inner cell mass; PriS, primitive streak; STB, syncytiotrophoblast; YSE, yolk sac endoderm. **f**, Volcano plot representing gene expression changes in LTR5Hs-CARGO versus nontarg-CARGO blastoid epiblast cells (using combined cells from epiblast and neo-epiblast clusters). Purple and light blue represent genes within 250 kb of an LTR5Hs, and the grey dots represent any other gene. Bold indicates genes further explored in Fig. 3b. The vertical dashed lines indicate statistically significant genes, fold change (FC) < −1.5 or FC > 1.5. The horizontal dashed line indicates −log_10_ of *P* = 0.05. DESeq2 Wald test false discovery rate (FDR) of 5%. *P* values were corrected for multiple testing using the Benjamini–Hochberg method^52^. Source Data

**Extended Data Fig. 3:**
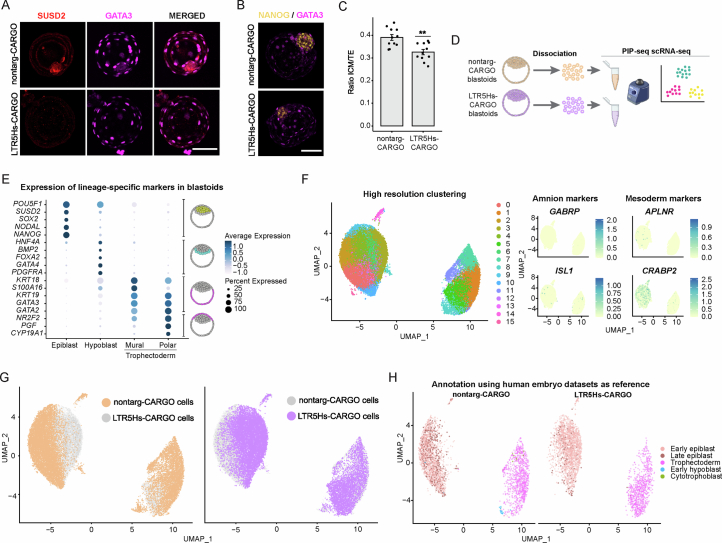
LTR5Hs-repression in human blastoids impairs lineage identity. **a**. Representative confocal images of a nontarg-CARGO and LTR5Hs-CARGO blastoid immunostained with antibodies against the epiblast marker SUSD2 (red) and the trophectoderm marker GATA3 (magenta), n = 3 blastoids from two independent biological replicates. White bar represents 100 µm. **b**. Representative confocal images of a nontarg-CARGO and LTR5Hs-CARGO blastoid immunostained with antibodies against the epiblast marker NANOG (yellow) and the trophectoderm marker GATA3 (magenta) n = 3, white bar represents 100 µm. **c**. Bar plots representing the ratio between the size of the inner cell mass (ICM) and the size of the trophectoderm cavity (TE) in nontarg-CARGO and LTR5Hs-CARGO blastoids. Each dot represents measures from tens of blastoids in each independent biological replicate (n = 11, 7 biological replicates but 4 of them with technical replicates). Unpaired two-tailed Student’s t-test, *P* = 0.0012. Data represents mean values +/− SEM. **d**. Schematic of particle-templated instant partition sequencing (PIP-seq) of single cells obtained from nontarg- or LTR5Hs-CARGO blastoid dissociation. The PIP-seq illustrations were created in BioRender. Fueyo, R. (2025) https://BioRender.com/3g5yuqw. **e**. Dot plot showing the expression of genes indicated on the y-axis in cells assigned to the clusters indicated on the x-axis. Cartoons on the right highlight the location of cells expressing the subset of genes in the human blastocysts/blastoids. **f**. High resolution clustering (resolution = 1) of PIP-seq results (left) and UMAP of the expression of amnion and mesoderm markers (right) in those clusters. **g**. UMAP of transcriptomes from single cells dissociated from blastoids and colored by their origin for better visualization of the UMAP depicted in Fig. 2d, nontarg-CARGO blastoids (left, orange) and LTR5Hs-CARGO (right, purple). **h**. UMAP representing the nontarg-CARGO and LTR5Hs-CARGO transcriptomes colored by the assigned human embryo transcriptomic counterpart using the computational tool published in ref. ^51^. Source Data

We next asked whether hypomorphic blastoids with medium levels of LTR5Hs repression show lineage defects. Immunostainings with epiblast (KLF17 and SUSD2), hypoblast (GATA4) and trophectoderm (GATA3) markers revealed that medium LTR5Hs-CARGO blastoids had a diminished number of KLF17^+^ and GATA4^+^ cells, and a decreased SUSD2 signal compared with nontarg-CARGO blastoids, suggesting defects in the epiblast and hypoblast lineages (Fig. 2a,b and Extended Data Fig. 3a). Of note, this decrease was not due to a loss of epiblast-like cells, as NANOG was detected in both nontarg-CARGO and LTR5Hs-CARGO blastoids (Extended Data Fig. 3b). By contrast, we detected an increased number of GATA3^+^ cells, accompanied by smaller inner cell mass to trophectoderm ratios (Fig. 2a,b and Extended Data Fig. 3c).

To systematically interrogate changes in gene expression and lineage allocation associated with LTR5Hs repression, we profiled transcriptomes of single cells by particle-templated instant partition sequencing (PIP-seq; Extended Data Fig. 3d and Supplementary Table 3). We selected two medium repression lines (circles outlined in black, Fig. 1d) and two nontarg-CARGO lines. We generated a global embedding of all samples amounting to 31,028 cells and 37,468 genes and performed cell cluster annotation based on markers of the blastocyst^18^ (Fig. 2c and Extended Data Fig. 3e). In parallel, we coloured cells based on their nontarg-CARGO or LTR5Hs-CARGO origin (Fig. 2d). Cells expressing markers of all blastocyst lineages were recovered in our analysis (Fig. 2c and Extended Data Fig. 3e). Within the trophectoderm lineage, we could distinguish mural and polar trophectoderm based on expression of *NR2F2*, *PGF* and *CYP19A1* in the latter^18,26,27^ (Extended Data Fig. 3e). Projection of the transcriptomes into a collection of human embryo datasets (Methods) confirmed that the nontarg-CARGO cells matched transcriptomes of the pre-implantation embryo (Fig. 2e, orange). Increased resolution of clustering failed to call amnion or mesoderm clusters. In agreement, amnion and mesoderm markers were either not expressed or did not overlap a specific cluster (Extended Data Fig. 3f).

**Extended Data Fig. 4:**
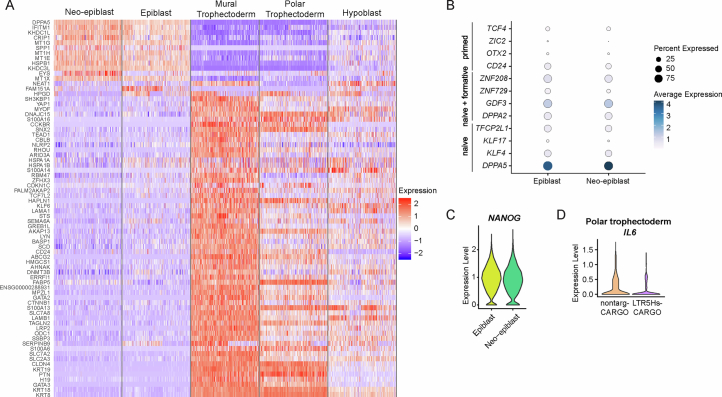
Lineage identity features of LTR5Hs-repressed blastoids. **a**. Heatmap representing the expression of genes identified as markers of the clusters indicated on top in the PIP-seq data. Color scale represents z-score. **b**. Dot plot showing the expression of naive, formative and primed pluripotency genes indicated on the y-axis in cells assigned to the epiblast or neo-epiblast clusters indicated on the x-axis. **c**. Violin plot of *NANOG* normalized expression levels in the epiblast and neo-epiblast clusters. **d**. Violin plot of *IL6* normalized expression levels in the polar trophectoderm of nontarg-CARGO and LTR5Hs-CARGO blastoids.

We observed the emergence of a new epiblast-adjacent cluster (dark green in Fig. 2c), which we refer to as ‘neo-epiblast’, by virtue of its transcriptional proximity to the epiblast. Although the neo-epiblast cells originated predominantly from the LTR5Hs-CARGO blastoids (purple in Fig. 2d and Extended Data Fig. 3g), the epiblast cluster was populated mostly by nontarg-CARGO cells, indicating that the neo-epiblast stems from gene expression changes driven by LTR5Hs repression. Projection of the LTR5Hs-CARGO cells into the human embryo reference datasets revealed they clustered with a less mature pre-implantation epiblast than the nontarg-CARGO epiblast-like cells (Fig. 2e and Extended Data Fig. 3h), suggesting that LTR5Hs repression affects epiblast maturation in blastoids. Nonetheless, HERVK LTR5Hs repression is not promoting a more pluripotent state, as the only naive pluripotency transcription factors changing upon LTR5Hs repression are *DPPA5* (higher expression) and *KLF17* (lower expression). We also detected a minor decrease in the primed pluripotency markers, consistent with the neo-epiblast cells being more immature (Extended Data Fig. 4a–c).

In addition to changes in the epiblast, we also observed a reduced allocation of LTR5Hs-CARGO cells to the hypoblast cluster, confirmed by the projections into the human embryo reference datasets (Fig. 2c–e and Extended Data Fig. 3g,h). The trophectoderm was the least affected by LTR5Hs repression overall, in agreement with its lowest HERVK expression. However, in contrast to the immunostainings, we did not detect an increased proportion of trophectoderm cells in the scRNA-seq analysis of the LTR5Hs-CARGO blastoids (see note in Methods). Beyond this caveat, we noticed a diminished contribution of the LTR5Hs-CARGO cells to the polar trophectoderm cluster and a decreased expression of *IL6*, a highly expressed interleukin in the polar trophectoderm that signals to the epiblast (Fig. 2c,d and Extended Data Fig. 4d). As maturation of the polar trophectoderm is dependent on the signals from the epiblast^17^, its decrease in the LTR5Hs-CARGO blastoids may be an indirect consequence of the defective epiblast.

**Extended Data Fig. 5:**
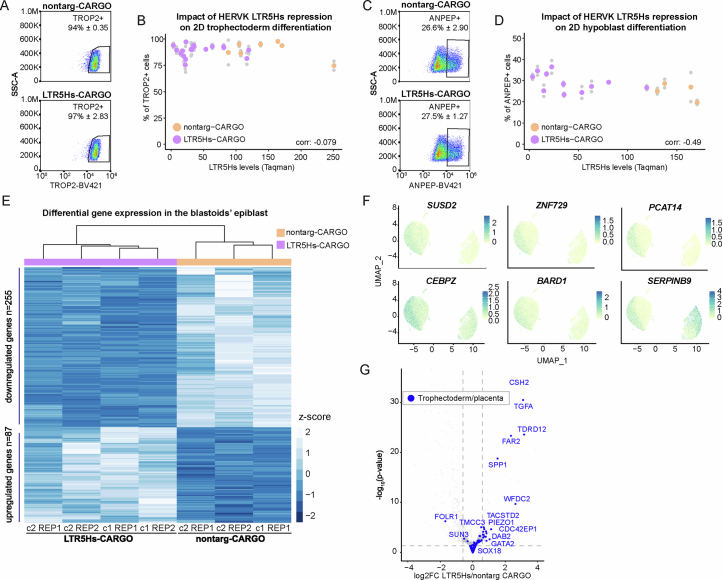
LTR5Hs regulates gene expression in the epiblast of blastoids. **a**. Flow cytometry density plots representing the analysis of TROP2+ cells compared to size scatter in 2D cultures of nontarg-CARGO (7 clonal cell lines in biological duplicates) and LTR5Hs-CARGO hnPSCs (15 clonal cell lines in biological duplicates) upon 3 days of differentiation using the trophectoderm protocol described in ref. ^29^. **b**. Representation of the quantitative results of the flow cytometry experiments described in **a**. Correlation represents Pearson’s. **c**. Flow cytometry density plots representing the analysis of ANPEP+ cells compared to size scatter in 2D cultures of nontarg-CARGO (4 clonal cell lines in biological duplicates) and LTR5Hs-CARGO hnPSCs (11 clonal cell lines in biological duplicates) upon 3 days of differentiation using the hypoblast protocol described in ref. ^55^. **d**. Representation of the quantitative results of the flow cytometry experiments described in **c**. Correlation represents Pearson’s. **e**. Heatmap and hierarchical clustering representing differentially expressed genes in the blastoids generated from nontarg-CARGO or LTR5Hs-CARGO hnPSCs cell lines (two clonal cell lines from each condition, n = 2 PIP- seq biological replicates per clone, except for nontarg-CARGO clone 1, n = 1). Color scale represents z-score. **f**. UMAP of the selected downregulated genes showing higher expression in the blastoids epiblast (left cluster) compared to the neo-epiblast cluster (right). **g**. Volcano plot representing gene expression changes in in LTR5Hs- versus nontarg-CARGO blastoid epiblast cells (using combined cells from epiblast and neo-epiblast clusters). Only genes from the trophectoderm or placenta lineages obtained from literature searches and refs. ^18,27^ are colored in dark blue, the rest of genes are grey color. Vertical dashed lines indicate fold change < −1.5 or > 1.5 and horizontal dashed line indicates -log10 of *P* = 0.05. DESeq2 Wald test FDR 5%. P-values are corrected for multiple testing using the Benjamini and Hochberg method^52^. Source Data

To test whether lineage defects were recapitulated in 2D hnPSCs cultures, we analysed the effect of LTR5Hs repression on direct trophectoderm and hypoblast differentiations. LTR5Hs repression did not change the potential of hnPSCs to differentiate into the trophectoderm (TROP2^+^ cells; Extended Data Fig. 5a and quantified in Extended Data Fig. 5b) or the hypoblast (ANPEP^+^ cells; Extended Data Fig. 5c and quantified in Extended Data Fig. 5d). Thus, the hypoblast defect that we observed in blastoids may be an indirect consequence of perturbed epiblast function or maturation. Overall, our results show that LTR5Hs repression disrupts lineage determination in human blastoids and results in a major change in the blastoids epiblast transcriptome.

## LTR5Hs regulates epiblast transcription in *cis*

To investigate transcriptome changes in the blastoids, we performed differential gene expression analysis between the nontarg-CARGO and the LTR5Hs-CARGO epiblast-like cells (Methods). We identified 255 and 87 transcripts that were downregulated and upregulated, respectively (Extended Data Fig. 5e and Supplementary Table 4). Only 122 and 24 of these genes significantly changed in the high and medium repression hnPSCs, respectively, highlighting distinct regulatory outputs of LTR5Hs in 2D hnPSCs cultures versus blastoids. LTR5Hs elements function as enhancers over distances of up to 250 kb from their target promoters^10^. We found that 84% of the downregulated transcripts in the epiblast lie within 250 kb of an LTR5Hs, compared with 1.62 Mb of median distance from all human protein-coding genes. This suggests direct regulation by LTR5Hs in *cis* (Fig. 2f). As expected, downregulated genes also display a decrease in expression when comparing neo-epiblast and epiblast clusters (Extended Data Fig. 5f).

The upregulated genes were typically not near LTR5Hs, suggesting indirect effects (Fig. 2f). However, many upregulated genes are associated with trophectoderm and/or placental development (Extended Data Fig. 5g). As examples, *CSH2* encodes for the lactogen hormone^28^; *GATA2* is a trophectoderm transcription factor^18^; *TACSTD2* and *DAB2* are trophoblast markers^18,29^; and, notably, *TGFA* increases blastocoel size in mouse^30^, suggesting that it could contribute to the larger trophectoderm compartment in the LTR5Hs-CARGO blastoids (Fig. 2a,b and Extended Data Fig. 5g). Together, these observations point to LTR5Hs elements regulating the blastoid epiblast genes in *cis*. This is in turn associated with an indirect upregulation of trophectoderm specification and maturation genes. Nonetheless, despite this upregulation, these cells remain closer to the naive pluripotent state in their overall transcriptome (Fig. 2e and Extended Data Fig. 4b).

## LTR5Hs elements are epiblast enhancers

We sought to address whether the effects on the epiblast gene expression were dependent on the LTR5Hs DNA sequence. To establish sequence dependency and rule out ectopic silencing in our KRAB–dCas9 experiments, we selected six LTR5Hs elements located in *cis* to genes downregulated in the neo-epiblast with potential or known functions in development (Extended Data Fig. 5f). For example, SUSD2 is a marker of human naive pluripotency^20,31,32^, ZNF729 of naive and formative pluripotency^32^, and BARD1 is a BRCA1 partner essential for embryonic development in mice^33^.

**Fig. 3:**
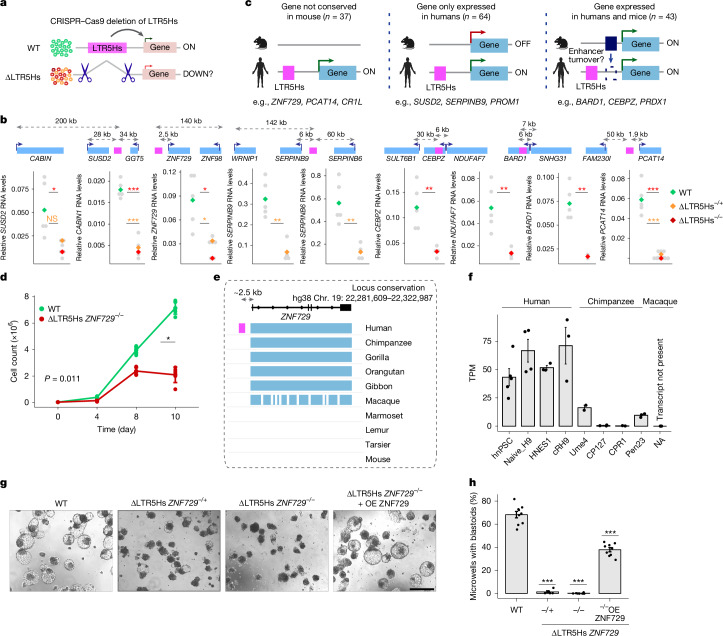
A human-specific LTR5Hs insertion is essential for blastoid formation. **a**, Strategy for testing LTR5Hs sequence dependency. WT, wild-type. **b**, RT–qPCR results of the indicated genes in wild-type or ΔLTR5Hs hnPSCs. RNA values were normalized to *RPL13A*. Above each plot, schematics of the locus are included. The grey dots represent expression in each clone, and the diamonds represent median values. *n* (independent clones) per gene: *SUSD2/CABIN*, WT = 5, heterozygous = 2 and homozygous = 5; *ZNF729*, WT = 5, heterozygous = 4 and homozygous = 2; *SERPINB9/6*, WT = 5 and heterozygous = 4; *CEBPZ/NDUFAF7*, WT = 5 and homozygous = 4; *BARD1*, WT = 5 and homozygous = 2; and *PCAT14*, WT = 5, heterozygous = 6 and homozygous = 6. Significance was determined by an unpaired, two-tailed Student’s *t*-test. The asterisk colour indicates homozygous (red) or heterozygous (yellow); specific *P* values are included as source data. NS, not significant. **c**, Expression and conservation of the LTR5Hs-regulated genes between humans and mice using data in ref. ^34^. The silhouettes of the human and mouse were created in BioRender. Fueyo, R. (2025) https://BioRender.com/tmgd0pn. **d**, Cell proliferation curves in wild-type (green) and ΔLTR5Hs *ZNF729*^−/−^ hnPSCs (red). *n* = 3 biological replicates. Data represent mean ± s.d. Significance was determined by one-way analysis of variance (ANOVA). **e**, Cartoon displaying the conservation of the gene *ZNF729* and its regulatory landscape across species. The LTR5Hs element is only present in humans. Data adapted from Cactus UCSC tracks^53,54^. Chr., chromosome. **f**, *ZNF729* expression in human^29,55,56^ and chimpanzee^39^ naive PSCs; rhesus monkey naive PSCs lack the transcript. Data are presented as mean ± s.e.m. Each dot represents a bulk RNA-seq biological replicate: hnPSC = 4, naive_H9 = 4, HNES1 = 3, cRH9 = 3, Ume4 = 2, CP127 = 3, CPR1 = 2 and Pen23 = 3. TPM, transcripts per million. **g**, Representative bright-field images of blastoids or dark spheres generated from wild-type hnPSCs, ΔLTR5Hs *ZNF729*^−/+^, ΔLTR5Hs *ZNF729*^−*/*−^ hnPSCs or ΔLTR5Hs *ZNF729*^−*/*−^ hnPSCs expressing a ZNF729 transgene (rescue, ΔLTR5Hs *ZNF729* + overexpression (OE) ZNF729). Scale bar, 400 µm. **h**, Quantification of blastoid formation efficiency from panel **g**. Data are presented as mean ± s.e.m. Each dot represents a biological replicate from one clone; we used three clones per condition in biological triplicates. Significance was determined by an unpaired, two-tailed Student’s *t*-test: *P* = 7.6 × 10^−10^ for *ZNF729*^−/+^, *P* = 4.47 × 10^−14^ for ΔLTR5Hs *ZNF729*^−*/*−^ and *P* = 7.63 × 10^−9^ for ΔLTR5Hs *ZNF729*  + OE ZNF729. Source Data

**Extended Data Fig. 6:**
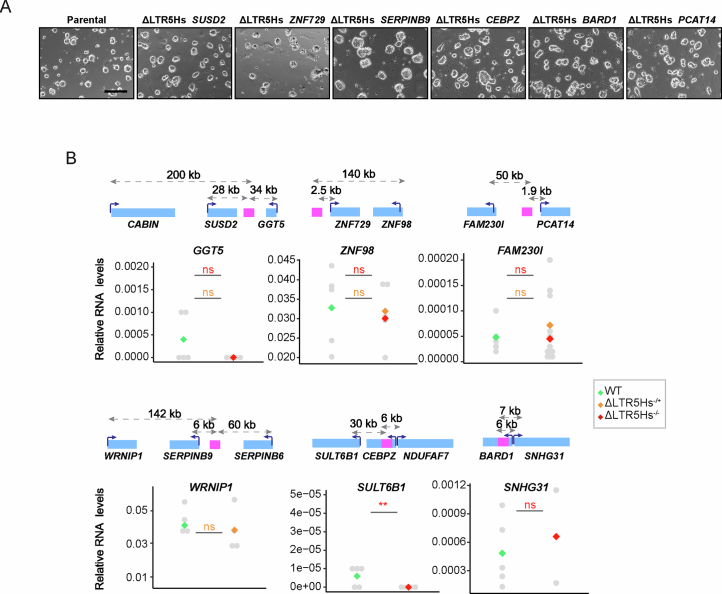
Expanded characterization of the ΔLTR5Hs hnPSCs. **a**. Bright field images of ΔLTR5Hs hnPSCs with the genes affected by each deletion indicated above each image. Black bar represents 200 µm. These images are representative of the cells morphology in culture, n = 2 biological replicates. **b**. RT-qPCR results of genes unaffected by LTR5Hs deletions at gene loci shown in Fig. 3b. Plots display the expression of the indicated genes in wild-type or ΔLTR5Hs hnPSCs. RNA values are normalized relative to *RPL13A*. Above each plot, schematic depiction of the locus is shown. Blue rectangles indicate genes, pink indicates the closest LTR5Hs element, the number on top of the dashed arrows displays distance from the promoter to the LTR5Hs. Grey dots represent expression values obtained in each clone, green, orange, and red squares represent median values. N (independent clones) per gene: *GGT5*: WT = 5, heterozygous=2, homozygous=5; *ZNF98*: WT = 5, heterozygous=4, homozygous=2; *WRNIP1*: WT = 5, heterozygous=4; *SULT6B1*: WT = 5, homozygous=4; *SNHG31*: WT = 5, homozygous=2; *FAM230I*: WT = 5, heterozygous=6, homozygous=6. Unpaired two-tailed Student’s t-test, asterisk color indicates homozygous (red) or heterozygous (yellow), specific p-values included as Source Data, ns: not significant. Source Data

We engineered hnPSCs in which each of the six LTR5Hs elements has been homozygously or heterozygously deleted, one at a time, in multiple clonal lines (Fig. 3a). We then measured the effects of each deletion on gene expression compared with wild-type cells that underwent clonal selection in parallel (oligos in Supplementary Table 1). In each case, we observed that LTR5Hs deletion led to downregulation of the candidate target gene (Fig. 3b). For *SERPINB9*, homozygous deletions could not be recovered. We also noticed a slower growth of the ΔLTR5Hs *ZNF729* clones, suggesting a role in proliferation. Apart from this, the clonal cell lines were morphologically undistinguishable from wild-type hnPSCs (Extended Data Fig. 6a).

In some cases, LTR5Hs deletion affected the expression of more than one gene (for example, *SUSD2*/*CABIN1*, *SERPINB9/SERPINB6* and *CEBPZ*/*NDUFAF7*; Fig. 3b), whereas in others, there was no effect on other genes located in *cis* (Extended Data Fig. 6b), suggesting selectivity in promoter responsiveness. For each deletion, we observed at least one unaffected gene, confirming that the deletions are not broadly altering the locus. Overall, promoters downregulated upon LTR5Hs deletions were located at various distances from LTR5Hs and represented different LTR5Hs–promoter orientations. Considering that dependence on DNA sequence, the ability to activate distally and independence of orientation are hallmarks of enhancers, we conclude that LTR5Hs elements function as hominoid-specific enhancers for many human epiblast genes.

## Hominoid transcriptome diversification

**Extended Data Fig. 7:**
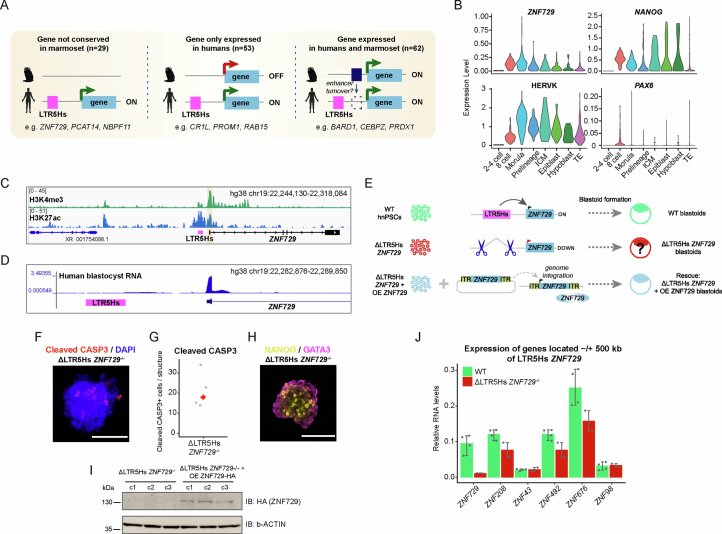
Diversification of the human epiblast transcriptome by LTR5Hs and characterization of the ΔLTR5Hs ZNF729 hnPSCs. **a**. Epiblast expression and evolutionary conservation of LTR5Hs-regulated genes in marmosets, data published in ref. ^34^. The silhouettes of the human and marmoset were created in BioRender. Fueyo, R. (2025) https://BioRender.com/tmgd0pn. **b**. *ZNF729* and HERVK normalized expression levels in human pre-implantation scRNA-seq data^18^. *NANOG* and *PAX6* are depicted as a reference for a highly or lowly expressed transcription factor, respectively. **c**. IGV browser capture showing ChIP-seq signal of H3K4me3 (top, green) and H3K27ac (bottom, blue) at the LTR5Hs *ZNF729* locus. **d**. UCSC browser capture of isoform resolved transcriptomic data in human blastocysts^98^ at the LTR5Hs *ZNF729* locus. **e**. Approach to address the essentiality of the human-specific LTR5Hs *ZNF729* in blastoid formation. Top section represents wild-type hnPSCs (green). Middle section shows ΔLTR5Hs *ZNF729* hnPSCs (red, i.e. LTR5Hs has been homozygously or heterozygously deleted). Bottom section represents the rescue experiment, performed by overexpressing ZNF729 (blue). **f**. Representative ΔLTR5Hs *ZNF729*^−*/*−^ dark sphere immunostained with cleaved-CASP3 (red) and DAPI (blue). White bar represents 100 µm. **g**. Quantification of cleaved-CASP3 immunostaining described in f (n = 5 stained dark spheres, two biological replicates). **h**. Representative ΔLTR5Hs *ZNF729*^−*/*−^ dark sphere immunostained with NANOG and GATA3, n = 3 biological replicates, white bar represents 100 µm. **i**. Western blot validating the expression of the ZNF729 protein from the integrated *ZNF729-HA* cDNA transgene in three clonal cell lines of ΔLTR5Hs *ZNF729* hnPSCs. Membrane blotted with antibodies against the HA tag and β-ACTIN as loading control blotted on the same membrane. N = 2 biological replicates. For gel source data, see Supplementary Fig. 1. **j**. RT-qPCR expression analysis of genes locates within ~1 Mb of the LTR5Hs *ZNF729* locus. Plot displays the expression of the indicated genes in wild-type (green, n = 4) or ΔLTR5Hs *ZNF729*^−/−^ (red, n = 2) hnPSCs. RNA values are normalized relative to *RPL13A*. Data represents mean values +/− SD. Source Data

We next asked whether LTR5Hs had a role in the hominoid-specific diversification of the epiblast transcriptome. We investigated the expression and conservation of LTR5Hs-regulated genes between humans, marmosets (a primate that lacks HERVK LTR5Hs) and mice. We focused on candidate direct gene targets of LTR5Hs, defined as those downregulated upon LTR5Hs repression in the epiblast of blastoids and located within 250 kb of an LTR5Hs. Then, we drew on the transcriptomes of staged-match human, marmoset and mouse pre-implantation embryos^34^. Out of the 144 LTR5Hs direct target genes, 37 lack an orthologue in mice and thus are not expressed, whereas 29 do not have an orthologue in marmoset (Fig. 3c, Extended Data Fig. 7a and Supplementary Table 5).

The remaining genes have a mouse or marmoset orthologue, and we analysed their expression in the pre-implantation epiblast of these species, classifying the genes as ‘expressed’ or ‘not expressed’ (Methods). Among the conserved genes, 64 were expressed in humans but not in mice, whereas 53 were expressed in humans but not in marmoset (Fig. 3c and Extended Data Fig. 7a). Thus, the *cis*-regulatory activity of LTR5Hs has contributed to the species-specific diversification of the epiblast transcriptome, with a 101 and 82 LTR5Hs-dependent genes being expressed in humans but not in mice or marmosets, respectively.

## An essential human-specific LTR5Hs insertion

Other orthologous genes showed conserved epiblast expression, despite being dependent on LTR5Hs in humans. This may reflect ‘transposon addiction’^35,36^, in which insertion of the strong LTR5Hs enhancer in hominoids may have led to turnover of the ancestral enhancer (or enhancers). Accordingly, we speculated that the LTR5Hs effect on development is more likely to arise from such transposon addiction than from young LTR5Hs elements regulating recently emerged genes. Contrary to this expectation, the only LTR5Hs deletion with an observable hnPSC phenotype was at the *ZNF729* locus. Indeed, ΔLTR5Hs *ZNF729*^−/−^ hnPSCs exhibited longer doubling times (34 h versus 19 h for wild-type cells; Fig. 3d). This was surprising, as this LTR5Hs insertion is unique to humans, and the *ZNF729* gene is also evolutionary young (Fig. 3e). Although the precise evolutionary age of KRAB zinc-finger genes is challenging to determine, previous analyses suggest that *ZNF729* emerged in the Old World anthropoids (*Catarrhini*) lineage^37,38^ (Supplementary Table 5).

In humans, *ZNF729* expression is robust and correlates with HERVK activity (Fig. 3f and Extended Data Fig. 7b). However, although *ZNF729* is present in the chimpanzee genome, it is poorly expressed in naive PSCs in the chimpanzee^39^. Moreover, unguided transcriptome assembly (Methods) uncovered no evidence for *ZNF729* expression in naive PSCs in the macaque^40^. This suggests that *ZNF729* expression during pre-implantation may be unique to humans and linked to the insertion of the LTR5Hs enhancer. Of note, in agreement with enhancer rather than alternative promoter function, this LTR5Hs element displays enhancer chromatin marks (high H3K27ac and low H3K4me3 levels) and *ZNF729* transcript reads originate exclusively from the promoter according to the long-read human embryo transcriptomes (Extended Data Fig. 7c,d).

Clonal cell lines lacking the LTR5Hs *ZNF729* element in heterozygosity (ΔLTR5Hs *ZNF729*^−/+^) or homozygosity (ΔLTR5Hs *ZNF729*^−/−^) failed to form blastoids and instead gave rise to dark spheres resembling those observed on en masse LTRHs repression (Fig. 3g,h and Extended Data Fig. 7e). These dark spheres also expressed cleaved CASP3, as well as NANOG and GATA3 at the edges (Extended Data Fig. 7f–h). Blastoid potential of the ΔLTR5Hs *ZNF729*^−/−^ hnPSCs was partially rescued by a transgene encoding *ZNF729* cDNA, indicating that ZNF729 mediates the phenotype (Fig. 3g,h, Extended Data Fig. 7e,i and Supplementary Fig. 1). The incomplete rescue is probably due to suboptimal transgene expression, rather than the regulation of multiple genes by the LTR5Hs insertion, as transcription of other genes at the locus is not affected by the LTR5Hs deletion (Extended Data Fig. 7j). Our results indicate that even highly species-specific retrotransposons can contribute to developmentally essential functions.

## ZNF729 binds to GC-rich sequences in hnPSCs

**Fig. 4:**
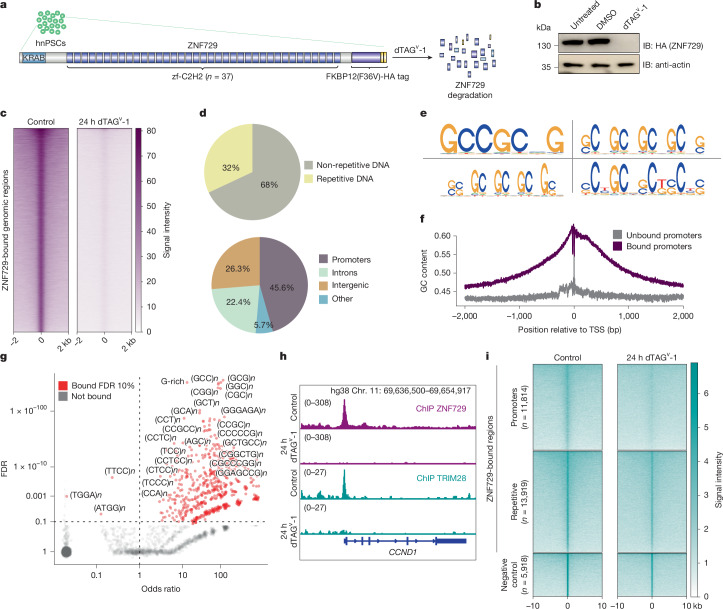
Widespread binding of ZNF729 at GC-rich sequences and promoters in hnPSC. **a**, Schematic depicting endogenous tagging of ZNF729, with an FKBPV(F36V) and two HA tags in hnPSCs (ZNF729–FH hnPSCs). Upon addition of dTAG^v^-1, ZNF729 is degraded. The protein structure was drawn with IBS 2.0 (ref. ^57^). **b**, Representative western blot of ZNF729–FH hnPSCs untreated, treated with DMSO as control or treated with dTAG^v^-1 for 24 h. The membrane was blotted with an antibody to the HA tag and with α-actin as the loading control blotted on the same membrane. For gel source data, see Supplementary Fig. 1. *n* = 2 biological replicates. IB, immunoblot. **c**, Heatmap displaying ZNF729–FH ChIP–seq signal over its bound genomic regions (*n* = 46,398) in DMSO treated (control) and 24-h dTAG^v^-1-treated ZNF729–FH hnPSCs (*n* = 2 biological replicates). **d**, Pie charts displaying the genomic features of ZNF729–FH-occupied regions. The pie charts show peak distribution between repetitive or non-repetitive DNA (top) and between promoters, intergenic or intronic DNA regions (bottom). **e**, Top four DNA sequence motifs obtained when performing motif discovery analysis on the ZNF729–FH ChIP–seq non-repetitive peaks using SeqPos^58^. **f**, Plot representing GC content at ZNF729–FH-bound (purple line) or unbound (grey line) promoters. TSS, transcription start site. **g**, Scatter plot displays the odds ratio of ZNF729–FH binding to simple repeats that contain either G or C or both. The vertical dashed line indicates an odds ratio of 1. The horizontal dashed line indicates FDR of 10%. Significantly bound (FDR of 10%) are depicted in red and not bound in grey. **h**, IGV genome browser capture of the ZNF729–FH ChIP–seq signal (top two tracks, purple) or the TRIM28 ChIP–seq signal (bottom two tracks, turquoise) in DMSO-treated (control) and 24-h dTAG^v^-1-treated ZNF729–FH. **i**, Heatmap displaying the TRIM28 ChIP–seq signal over ZNF729-bound non-repetitive promoters (top), the repetitive regions (middle) or the regions bound by TRIM28 that do not overlap with ZNF729 (bottom). The ChIP–seq signals from control-treated (left) or dTAG^v^-1-treated (right) ZNF729–FH hnPSCs are shown (*n* = 2 biological replicates).

**Extended Data Fig. 8:**
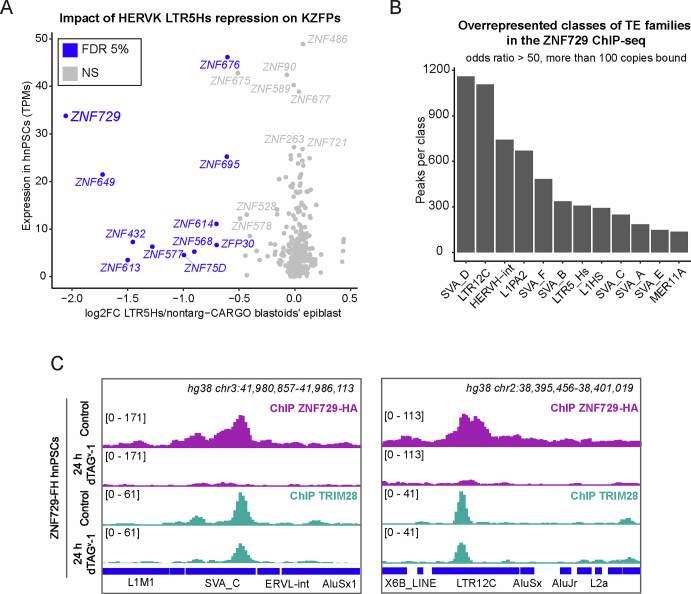
ZNF729 binds transposable elements in hnPSCs. **a**. *ZNF729* is the most dysregulated KZFP in the blastoids epiblast upon LTR5Hs-repression. Scatter plot depicting expression changes in KZFPs (curated in Krabopedia^99^) in the blastoids epiblast upon LTR5Hs repression (x-axis) vs their expression in hnPSCs (y-axis). FDR 5%. **b**. Histogram representing overrepresented classes of transposable elements (TEs) in the ZNF729-FH bound DNA regions. Only the top 12 families are represented. Y-axis displays the number of bound TEs from each family. **c**. IGV genome browser capture of ZNF729-FH ChIP-seq signal (top two tracks, purple) or TRIM28 ChIP-seq signal (bottom two tracks, turquoise) in DMSO treated (control) and 24 h dTAG^v^-1 treated ZNF729-FH at loci containing TEs bound by ZNF729 (SVA_C, left panel, hg38 chr3:41,980,857-41,986,113; LTR12C, right panel hg38 chr2:38,395,456-38,401,019).

Structurally, ZNF729 consists of a KRAB repressor domain and the highest number of zinc-finger domains in the human proteome: 37 (Fig. 4a). In addition, *ZNF729* is the most downregulated KRAB zinc-finger protein (KZFP) in the epiblast of the LTR5Hs-CARGO blastoids (Extended Data Fig. 8a). To acutely perturb ZNF729 function, we derived homozygous clonal cell lines with *ZNF729* endogenously tagged with a dTAG-inducible degron and two haemagglutinin (HA) tags (Fig. 4a). We confirmed that upon dTAG^v^-1 addition, ZNF729–FKBP–HA (ZNF729–FH) was degraded (Fig. 4b and Supplementary Fig. 1). HA chromatin immunoprecipitation followed by sequencing (ChIP–seq) of ZNF729–FH in control and dTAG^v^-1-treated conditions identified 46,398 bound regions; more than 95% of these peaks were lost with dTAG^v^-1 treatment, indicating specificity (Fig. 4c,h and Supplementary Table 6).

KZFPs bind to and repress transposable elements via recruitment of TRIM28 (also known as KAP1) and epigenetic repressors^41^. However, unlike most KZFPs, ZNF729 binds mostly to non-repetitive DNA, with almost 46% of the peaks overlapping promoters (Fig. 4d). Within transposable elements, ZNF729 binds to young transposable elements such as SVA_D/F/B or LTR5Hs itself (Extended Data Fig. 8b,c). Motif discovery analysis at the non-repetitive peaks varied among tools, but all recovered motifs rich in G and C, with diverse spacing and different lengths (SeqPos examples in Fig. 4e and Supplementary Table 7). We wondered whether the binding of ZNF729 at promoters can be explained by their high GC content in mammals. Indeed, GC content was much higher at ZNF729-bound than at unbound promoters (Fig. 4f). The discovery of GC-rich motifs could be a consequence, rather than a cause of ZNF729 binding at the promoters. However, we also observed binding of ZNF729 at simple repeats rich in G and/or C, even in the absence of association with promoters (Fig. 4g), suggesting a propensity of ZNF729 for binding GC-rich sequences.

We next tested whether the KZFP partner TRIM28 colocalizes with ZNF729. ChIP–seq of TRIM28 in control ZNF729–FH hnPSCs confirmed colocalization of both proteins genome wide (Fig. 4h,i). Noticeably, repetitive regions exhibited higher TRIM28 levels compared to promoters, consistent with multiple KZFPs probably binding and recruiting TRIM28 to repeats (Fig. 4i and Extended Data Fig. 8c). Degradation of ZNF729 affected TRIM28 genomic binding, diminishing the number of peaks from 17,460 to 3,160. This impact was less severe at repetitive regions, again consistent with potential KZFPs redundancy. By contrast, TRIM28 binding was strongly diminished at promoters, and TRIM28 regions not overlapping with ZNF729 were unaffected (Fig. 4i). This indicates that ZNF729 recognizes GC-rich sequences, including thousands of promoters where it recruits TRIM28.

## ZNF729 controls basic cellular functions

**Fig. 5:**
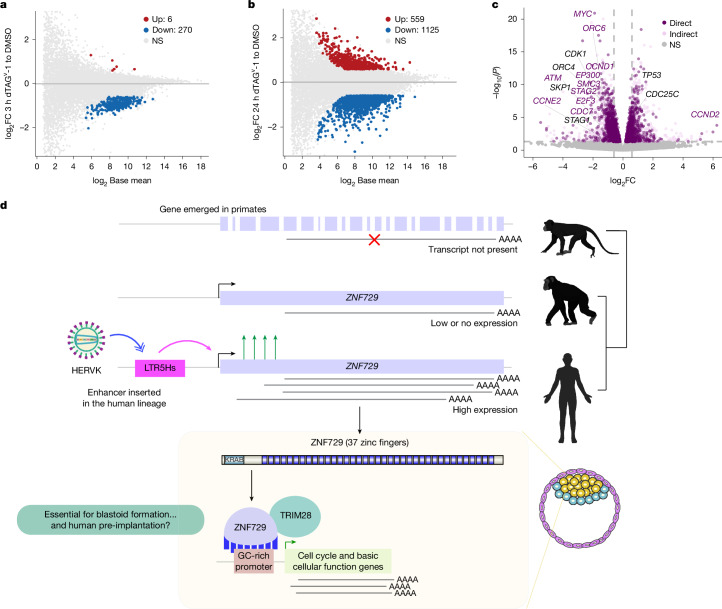
ZNF729 is a transcriptional regulator of basic cellular functions in hnPSCs. **a**, Logged intensity ratio (M) versus mean logged intensities (A) (MA) plot representing bulk RNA-seq results of ZNF729–FH hnPSCs treated with DMSO (control) or with dTAG^v^-1 for 3 h (*n* = 4). The *y* axis shows gene expression fold changes in 3-h dTAG^v^-1-treated versus DMSO-treated ZNF729–FH hnPSCs. FDR of 5%. The horizontal line indicates fold change of 0. **b**, MA plot representing bulk RNA-seq results of ZNF729–FH hnPSCs treated with DMSO (control) or with dTAG^v^-1 for 24 h (*n* = 4). The *y* axis shows gene expression fold changes in 24-h dTAGv-1-treated versus DMSO-treated ZNF729–FH hnPSCs. The horizontal line indicates fold change of 0. **c**, Volcano plot of gene expression changes in bulk RNA-seq analyses from ZNF729–FH hnPSCs treated with DMSO (control) or with dTAG^v^-1 for 24 h (*n* = 4), coloured by the presence (dark purple) or absence (light pink) of ZNF729–FH binding at the gene promoter. The labelled dots represent genes involved in the cell cycle according to gene set enrichment analysis-curated gene sets. Significance was determined using DESeq2 Wald test, FDR of 5%. *P* values were corrected for multiple testing using the Benjamini–Hochberg method^52^. **d**, Model for human-specific gain of function of ZNF729–FH in early development, facilitated by the insertion of HERVK LTR5Hs. The silhouettes of the human, chimpanzee and macaque were created in BioRender. Fueyo, R. (2025) https://BioRender.com/tmgd0pn.

**Extended Data Fig. 9:**
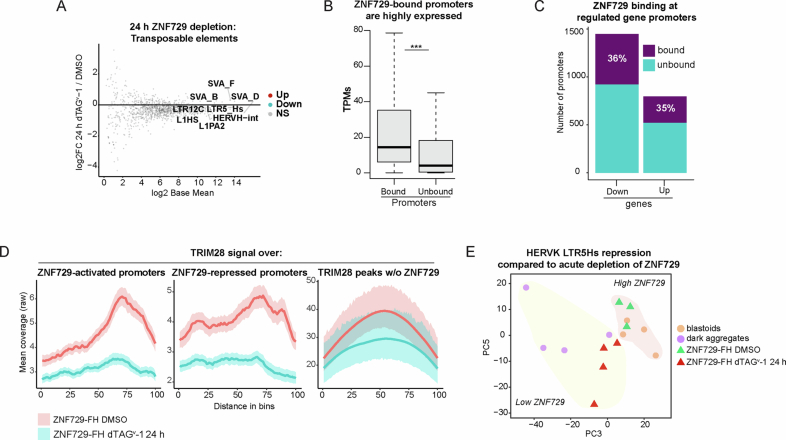
ZNF729 exerts activator or repressor function at target genes. **a**. Low impact of ZNF729-FH depletion on the expression of TEs. MA plot represents TEtranscript^87^-obtained data quantified with DESeq2^83^. Top ZNF729-bound TE families are labeled. **b**. Expression from gene promoters bound or unbound by ZNF729. Box plots show basal hnPSC expression (in TPMs) of genes bound or unbound by ZNF729, n = 4 biological replicates. Boxes show the median and the quartile 1 (Q1) to quartile 3 (Q3) range (interquartile range, IQR). Whiskers minimum corresponds to Q1 − 1.5 × IQR, and the maximum to Q3 + 1.5 × IQR. N = 461 bound promoters and n = 13883 unbound promoters. Two-sided Wilcoxon test, *P* < 2.2e-16. **c**. Stacked bar plot represents number of down- or upregulated genes upon ZNF729-FH depletion for 24 h with dTAG^v^-1 and the percentage of them directly bound by ZNF729 at their promoters (−1kb/+200 bp around the TSS). **d**. Metagene plots showing TRIM28 ChIP-seq signal over promoters activated or repressed by ZNF729 and over a negative control (TRIM28 bound regions not overlapping with ZNF729). Red represents ZNF729FH DMSO (control) and green dTAG^v^-1 treated. **e**. PCA analysis of bulk transcriptomes from blastoids, dark spheres formed upon high LTR5Hs repression, and DMSO-treated or dTAG^v^-1 treated ZNF729-FH hnPSCs. Red shade represents “normal / high” levels of ZNF729 (blastoids, and DMSO treated ZNF729-FH cells). Yellow shade represents “low” ZNF729 levels (dark spheres with high LTR5Hs repression, and dTAG^v^-1 treated ZNF729-FH hnPSCs). Note that high ZNF729 and low ZNF729 samples separate along PC3.

To investigate whether ZNF729 regulates gene expression, we treated ZNF729–FH hnPSCs with dTAG^v^-1 for 3 h or 24 h and performed RNA-seq. Differential gene expression analysis at 3 h detected mostly gene downregulation (Fig. 5a). Even at 24 h, there was a larger proportion of downregulated genes (Fig. 5b). Transposable element expression was not affected by loss of ZNF729 (Extended Data Fig. 9a), consistent with potential KZFP redundancy and persistence of TRIM28 at transposable elements (Fig. 4i).

We next asked whether transcriptomic changes occurred at ZNF729-bound promoters. We defined directly regulated genes as those significantly misregulated upon loss of ZNF729 and bound by ZNF729 within −1 kb or +200 bp from the transcription start site. Of downregulated and upregulated promoters, 36% and 35%, respectively, were directly bound, indicating that ZNF729 can act as a transcriptional activator or repressor (Extended Data Fig. 9b,c). TRIM28 occupancy at these promoters upon depletion of ZNF729 shows a clear reduction of the TRIM28 signal, albeit the shapes of the profiles differ between the ZNF729-activated and ZNF729-repressed targets, suggesting that different protein complexes may be driving these distinct outputs (Extended Data Fig. 9d).

Housekeeping promoters are typically GC rich and highly and broadly active. ZNF729-bound promoters are GC rich and also display high expression (Extended Data Fig. 9b). Ontology analysis of genes regulated by ZNF729 at 3 h are associated with basic cellular processes such as cell division, GTPase activity, RNA metabolic processes and others (Supplementary Table 8). This led us to reason that the ΔLTR5Hs *ZNF729*^−/−^ slow growth could be explained by cell-cycle genes affected upon depletion of ZNF729. Indeed, classic cell-cycle regulators such as *MYC*, *CDK1* or *CCND1* are bound and activated by ZNF729 (Figs. 4h and 5c). These results suggest that ZNF729 may be a major driver of phenotypes observed upon global LTR5Hs repression. Consistently, PCA revealed similarities between transcriptomes of ZNF729-depleted hnPSCs and those of high LTR5Hs repression dark spheres (Extended Data Fig. 9e).

## Discussion

Our results demonstrate how an evolutionary young gene, encoding a KZFP, gained expression in the human lineage upon capturing a retrovirus-derived enhancer. The product of the gene subsequently influenced essential cellular programs by binding and regulating GC-rich promoters (Fig. 5d).

We have shown that HERVK LTR5Hs activity is required for blastoid formation and lineage identity. LTR5Hs elements function as enhancers, and consequently, LTR5Hs repression severely affects the transcriptome of the epiblast of blastoids. LTR5Hs repression also leads to diminished hypoblast and polar trophectoderm cell numbers. Specification of these lineages in the embryo depends on the epiblast^17,22^, suggesting that these effects may originate in the defective epiblast. Alternatively, as described in mice, the hypoblast could be dedifferentiating back to the inner cell mass^42^.

The essentiality of HERVK LTR5Hs for blastoid formation probably stems from multiple mechanisms. One explored here in detail is the *cis*-regulatory activity of the LTR5Hs at the *ZNF729* locus, but other LTR5Hs enhancers and their target genes may also be required for human development. Moreover, a subset of HERVK LTR5Hs insertions encodes retroviral proteins that can assemble viral-like particles in human blastocysts^9,43^. Although HERVK retroviral proteins do not rescue the failed blastoid formation, they may still have other vital roles in the embryo^44^. Of note, LTR10A and MER41B elements also exert *cis*-regulatory functions of placental genes in trophoblast stem cells, suggesting that other retrotransposon families may also be essential in embryogenesis^45^.

We revealed a requirement for the human-specific LTR5Hs *ZNF729* for proliferation and blastoid formation potential. ZNF729 confers this essentiality by binding and regulating hundreds of crucial genes due to its affinity for GC-rich sequences, which are extremely abundant in mammalian housekeeping gene promoters. ZNF729 contains an intact KRAB domain, capable of mediating repression in a screen of transcriptional effectors^46^. In agreement, we showed that ZNF729 is the major transcription factor responsible for recruiting TRIM28 to promoters in hnPSCs. However, many regulated promoters are activated, rather than repressed, by ZNF729, an uncommon finding for a KZFP, albeit not a unique case^47^. Although the mechanism underlying this activation remains to be established, we note that TRIM28 has also been implicated in gene activation through mediating RNA polymerase II (RNAPII) pause–release^48–50^. Alternatively, ZNF729 activator function may be mediated by a heretofore unknown TRIM28-independent mechanism.

We wondered why an evolutionary young gene, controlled by a human-specific enhancer, is important for regulation of ancient cellular programs. We speculate that during primate evolution, the expansion of the zinc-finger array and mutations at amino acids contacting the DNA, ultimately resulted in a high affinity of ZNF729 for GC-rich sequences. In turn, this targeted ZNF729 to housekeeping promoters, potentially boosting their activity and providing a competitive proliferative advantage to cells expressing ZNF729. However, such regulation could have also led to a relaxation of the constraint on the ancient mechanisms controlling expression of proliferation genes, with ZNF729 ultimately supplanting one of such mechanisms and becoming essential for pre-implantation. These observations suggest that evolutionary remodelling of gene-regulatory networks can result not only in species-specific innovation but also create new dependencies and bestow essentiality on recently emerged *cis*-regulatory elements and genes.

## Methods

### Ethics

This work was performed following the 2021 ISSCR Guidelines^59^. The use of blood-derived induced naive pluripotent stem cells for the experiments described in this article was approved by the Stanford Stem Cell Research Oversight committee (SCRO protocol number 900).

### Cell culture

Induced hnPSCs were generated from peripheral blood cells by overexpressing NANOG and KLF2 by Masaki et al.^60^; the official name of this cell line is PB004. Cells have been authenticated by STR. Cells were grown on irradiated Cf1 mouse embryonic fibroblast (MEF) feeder layers (A34181, Fisher Scientific). Before experiments entailing next-generation sequencing, hnPSCs were plated without MEFs (feeder-free conditions) using Geltrex (A1413301, Gibco) as a matrix. hnPSCs were grown in PXGL^20^. This medium consists of N2B27 as base, which is made by mixing 1:1 DMEM/F-12 (D8437, Sigma) and Neurobasal (21103049, Thermo) with the following added supplements: 2 mM l-glutamine (25030024, Thermo), 100 µM 2-mercaptoethanol (M3148, Sigma), N2 and B27 supplements (17502048 and 7504044, Gibco) and 1× antibiotic–antimycotic solution (A5955, Sigma-Aldrich). To make PXGL, we freshly supplemented the following chemicals: 1 µM PD0325901 (S1036, Selleckchem), 2 µM XAV939 (SM38-200, Cell Guidance Systems), 2 µM Go 6983 (2285, Bio-Techne), 10 ng ml^−1^ recombinant human LIF (300-05, Preprotech) and 1 µg ml^−1^ of doxycycline to sustain NANOG and KLF2 transgenes expression in hnPSCs. Doxycycline was eliminated from the media for the nontarg-CARGO or LTR5Hs-CARGO induction experiments. Cells were passaged using TrypLE Express (12-605-010, Fisher Scientific) every 3–4 days or whenever colonies were too confluent. The cell incubator was kept at 37 °C and humidified at 7% CO_2_ and 5% O_2_ (hypoxia). All cell lines were tested monthly for *Mycoplasma*. For KRAB–dCas9 and ZNF729 (rescue) inductions, we used 2× water soluble cumate (QM150A-1, System Biosciences).

### Derivation of nontarg-CARGO and LTR5Hs-CARGO hnPSCs

hnPSCs were nucleofected using a Lonza 4D-Nucleofector using the P3 Primary X Kit-S (V4XP-3032, Lonza) with the DN100 program. Per nucleofection, we used 400,000 cells without MEF depletion. To generate the KRAB–dCas9 hnPSCs, 0.8 µg of a piggyBac construct containing KRAB–dCas9 under a cumate-inducible promoter^61^, the cumate repressor CymR and a puromycin selection cassette were co-nucleofected with 0.2 µg of the super piggyBac transposase (PB210PA-1, System Biosciences). Clones containing the integration were selected with puromycin (0.5 µg ml^−1^) for three passages. KRAB–dCas9 hnPSCs cells were later nucleofected with 0.8 µg of the piggyBac constructs containing nontarg-CARGO (#191319, Addgene)^10^ or LTR5Hs-CARGO (#191316, Addgene)^10^ and a neomycin selection cassette and 0.2 µg of the super piggyBac transposase. Cells were then selected with 200 µg ml^−1^ of G418 for 10 days. Cells (*n* = 2,000) were subsequently plated in a 10-cm^2^ plate containing MEFs and fed every day. On days 8–9, sparse colonies were visible and were picked and expanded for the experiments. Cells were treated with puromycin and G418 every few passages to sustain proper KRAB–dCas9 and CARGO array expression, as we noticed these transgenes get silenced over the passages in hnPSCs. For the ‘orthogonal repression of LTR5Hs’ experiments, a distinct array of gRNAs targeting LTR5Hs was designed and cloned into piggyBac using CARGO^23^ (gRNA sequences are in Supplementary Table 1). The LTR5Hs-Ortho-CARGO hnPSCs were generated as described above, with the only difference that this time the KRAB–dCas9 transgene was under a cumate-inducible *Ef1a* promoter to ensure high repression at the population level. Analysis of the role of the HERVK proteins in the dark spheres phenotype (‘rescue with HERVK ORFs’ experiment) was performed by selecting three high-repression LTR5Hs-CARGO clones that were previously demonstrated to give rise to dark spheres, and integrating into them multiple copies of a piggyBac transgene encoding a tagBFP and the proteins gag, pro and pol^24^ under a constitutive *Ef1a* promoter to ensure robust expression. High-repression LTR5Hs-CARGO hnPSCs positive for tagBFP were isolated and utilized for blastoid formation under cumate treatment to induce LTR5Hs repression.

### Genetic deletion of LTR5Hs and ZNF729 overexpression

Selected LTR5Hs elements were deleted from the genome using pairs of gRNAs designed using Benchling (Biology Software, 2023; Supplementary Table 1). crRNAs were purchased from IDT with the XT modification for stability. Cells (*n* = 400,000) were nucleofected with a ribonucleoprotein complex containing 1.65 µg of HiFi Cas9 Nuclease V3 (1081059, IDT) and 0.85 µl of a 1:1 ratio of 100 µM annealed tracRNA and crRNA. Cells were passaged once and then 2,000 cells were plated on a 10-cm^2^ plate with MEFs for colony picking. Clones were genotyped using PCR and Sanger sequencing, and heterozygous and homozygous clones were kept for experiments. For the rescue experiments in Fig. 3g,h, 400,000 ΔLTR5Hs *ZNF729*^−/−^ hnPSCs were nucleofected with a piggyBac plasmid subcloned from a pcDNA3 vector, containing ZNF729–HA cDNA (purchased from Genscript) and a puromycin selection cassette. Super piggyBac transposase was co-nucleofected. Cells were selected with 0.5 µg ml^−1^ puromycin for 10 days and ZNF729–HA expression was tested by western blot.

### Derivation of ZNF729–FKBP(F36V)–HA hnPSCs

To endogenously tag *ZNF729*, we performed homology-directed repair at the locus with a donor DNA providing the FKBP(F36V)^62,63^ and HA tags. To this end, we drew upon a previously published method^64^ based on the combination of Cas9 ribonucleoproteins and delivery of the donor template by AAV6 viral vectors. To generate the AAV viral particles, 2 × 15 cm^2^ dishes of 293FT cells (R70007, Invitrogen) at 60% confluency were transfected. The day of transfection, the 293FT cells ‘complete cell media’ (DMEM/high-glucose medium (SH30243.FS, Cytiva), 10% FBS (100-106, GeminiBio), 1X non-essential amino acids (1114-0050, Gibco), 1X GlutaMAX (4109-0036, Gibco) and 1X antibiotic–antimycotic (1524-0062, Gibco)) was refreshed 6 h before transfecting. Transfection was carried out using 120 µg polyethylenimine (PEI) per 15-cm^2^ plate, 22 µg of pDGM6 (#110660, Addgene)^65^ and 6 µg of AAV template (cloned in the pAAV-GFP backbone; #32395, Addgene)^66^. After 24 h, the medium was changed to ‘slow growth media’ (same as complete media, but with 2% FBS instead of 10%), and upon further 48 h of culture, the AAV viral particles were purified using one reaction of the AAVpro kit (6675, Takara Bio) and stored at −80 °C. The crRNA utilized to target the *ZNF729* C-terminal region was purchased from IDT with the XT modification for stability. Wild-type hnPSCs (*n* = 400,000) were nucleofected with the ribonucleoprotein complex containing 1.65 µg of HiFi Cas9 Nuclease V3 (1081059, IDT) and 0.85 µl of a 1:1 ratio of 100 µM annealed tracRNA and crRNA. Cells were seeded in a plate containing MEFs, PXGL, the ROCK inhibitor Y-27632 and the AAV viral particles containing the donor template. Media were changed after 24 h. Cells were passaged once and then 2,000 cells were plated on a 10-cm^2^ plate with MEFs for colony picking. Correct editing was analysed by PCR, Sanger sequencing and western blot. ZNF729 depletion was obtained upon addition of 500 nM of dTAG^v^-1 (6914, Tocris) for the indicated times.

### Blastoid formation

To generate blastoids, the protocol described in Kagawa et al.^17,67^ was followed with minor changes. hnPSCs were grown on MEFs and dissociated the day of the experiment into single cells. MEFs were depleted by culturing the dissociated cells in PXGL over a gelatin matrix (G1393, Sigma-Aldrich) for 1 h. We used 24-well Aggrewell 400 (34415, StemCell Technologies) plates as vessels. Upon multiple tests, we determined that starting from 76 cells per intended blastoid was optimal, so 91,200 hnPSCs were plated per well of the microwell plate (76 × 1,200 microwells). On the day of plating, cells were cultured in N2B27 base medium containing 10 µM Y-27632 (72304, StemCell Technologies). After 20–24 h, medium was changed to PALLY medium (N2B27 base medium supplemented with PD0325901 (1 µM), A83-01 (1 µM; HY-10432, MedChemExpress), 1-oleoyl lysophosphatidic acid sodium salt (LPA; 500 nM; 3854, Tocris), hLIF (10 ng ml^−1^) and Y-27632 (10 µM)). PALLY medium was refreshed the next day. Seventy-two hours after plating, medium was replaced with medium containing 500 nM of LPA. At 96 h, structures were collected and analysed as needed. In those experiments in which 2× cumate was added to induce KRAB–dCas9 expression, the drug was added on the day of plating concomitantly with cell aggregation.

### hnPSCs differentiation towards the trophectoderm lineage

Trophectoderm monolayer differentiation was completed as described previously^29,68^. In brief, hnPSCs were washed with PBS and then incubated with TrypLE Express for 10 min at 37 °C. Dissociated cells were washed in DMEM/F-12 (11-330-057, Thermo Fisher Scientific) with 0.1% Bovine Albumin Fraction V (15260037, Thermo Fisher Scientific) and resuspended in nTE-1 media (N2B27 media supplemented with 2 µM PD325901, 2 µM A83-01 and 10 ng ml^−1^ BMP4 (314-BP-010, R&D Systems)). Cells were counted and seeded to plates coated with 0.15 µg cm^−^^2^ laminin511-E8 (AMS.892 021, Amsbio) at a density of 2 × 10^4^ cells per cm^2^. Twenty-four hours after plating, media were changed to nTE-2 media (N2B27 media supplemented with 2 µM PD325901, 2 µM A83-01 and 1 µg ml^−1^ JAK inhibitor I (74022, StemCell Technologies)). Forty-eight hours after plating, the media were again changed to fresh nTE-2 media. To repress LTR5Hs elements during the differentiation, media were supplemented with 2× water-soluble cumate. Differentiations took place under hypoxic conditions.

### hnPSCs differentiation towards the hypoblast lineage

Hypoblast monolayer differentiation from hnPSCs was completed as previously described^55^. In brief, hnPSCs were washed with PBS and then incubated with TrypLE Express for 10 min at 37 °C. Dissociated cells were washed in DMEM/F-12 with 0.1% Bovine Albumin Fraction V and resuspended in a six-factor ‘6 F media’ (N2B27 media supplemented with 25 ng ml^−1^ FGF4 (100-31, PeproTech; stabilized with 1 µg ml^−1^ heparin sodium), 10 ng ml^−1^ recombinant human BMP4, 10 ng ml^−1^ recombinant human PDGF-AA (100-13A, Peprotech), 1 µM XAV939 (SM38-10, Cell Guidance Systems), 3 µM A83-01 (HY-10432, MedChem Express) and 0.1 µM retinoic acid (R2625, Sigma-Aldrich). Cells were counted and seeded to plates coated with 0.15 µg cm^−^^2^ laminin511-E8 at a density of 5 × 10^4^ cells per cm^2^. Twenty-four hours after plating, the medium was replaced with fresh 6F media. Forty-eight hours after plating, the medium was changed to a seven-factor ‘7F media’, which includes the same factors used in the 6F media, with the addition of 10 ng ml^−1^ recombinant human IL-6 (200-06, PeproTech). To repress LTR5Hs elements during the differentiation, media were supplemented with 2× water-soluble cumate. Differentiations took place under hypoxic conditions and flow cytometry measures were taken on day 3.

### Flow cytometry

After 3 days of trophectoderm or hypoblast differentiation, 200,000 cells were used for staining. Cells were pelleted and resuspended in 100 µl of N2B27 supplemented with 10 µM Y-27632 and either a 1:100 dilution of TACTSD2-BV421 for the trophectoderm differentiations (563243, BD Biosciences) or 1:200 dilution of ANPEP-BV421 for the hypoblast differentiations (301716, BioLegend). Cells were incubated on ice in the dark for 1 h and then washed twice with N2B27 supplemented with 10 µM Y-27632. Flow cytometry was performed on the SONY MA900 cell sorter and data were analysed using FlowJo (v10.10.0).

### RNA extraction and RT–qPCR

RNA extraction was performed using TRIzol (15596018, Life Technologies) directly on dissociated hnPSCs carrying the indicated perturbation or, in the case of blastoids and dark spheres, before RNA extraction, the structures were dissociated in a 1:1 mixture of trypsin-EDTA 0.5% (15-400-054, Fisher Scientific) and Accutase (07920, StemCell Technologies) for 5 min, diluted in N2B27 and spun down. Extraction was followed by RNA purification using a Direct-zol RNA-prep kit (R2052, Zymo Research) with DNAse treatment. Of RNA, 1 µg was retrotranscribed into cDNA using a SensiFAST cDNA synthesis kit (BIO-65053, Bioline), cDNA was diluted 1:4 with molecular grade water and 2 µl of this dilution was used for quantitative PCR (qPCR) with primers for each amplicon (Supplementary Table 1). qPCR was performed in a LightCycler 480 Instrument (II) using a SensiFAST SYBR (Bioline, BIO-98020). For experiments using Taqman probes, qPCR Primetime probes were purchased from IDT (sequences in Supplementary Table 1) and were combined for qPCR with the LightCycler 480 Probes Master mix (04707494001, Roche).

### CUT&RUN

Protocol was performed according to Meers et al.^69^ and using the CUTANA reagents from EpiCypher (concanavalin A-conjugated paramagnetic beads (21-1401), pAG-Tn5 (15-1017) and *Escherichia coli* spike-in DNA (18-1401)). We used 500,000 nontarg-CARGO or LTR5Hs-CARGO hnPSCs per condition. Cells were permeabilized using 0.005% of digitonin, and 0.5 µg of H3K9me3 primary antibody was used per sample (Supplementary Table 1). DNA was extracted using phenol–chloroform, and library preparation was performed using the NEBNext Ultra II Library Prep kit (E7645S, New England Biolabs). Libraries were sequenced paired-end for 150 cycles in a Novaseq 6000 Illumina sequencer.

### ChIP–seq, ChIP–seq libraries construction and sequencing

Cells were grown on feeder-free conditions using Geltrex to minimize MEF contamination in the sequencing. One 10 cm^2^ (approximately 6 × 10^6^ hnPSCs) was used per ChIP. Cells were crosslinked in PBS containing 1% methanol-free formaldehyde (28908, Pierce) for 10 min. Fixation was quenched during 10 min by adding a final concentration of 0.1 M of glycine. Upon harvesting, cells were resuspended in buffer 1 (50 mM HEPES-KOH pH 7.5, 140 mM NaCl, 1 mM EDTA, 10% glycerol, 0.5% NP-40 and 0.25% Triton X-100) and incubated for 10 min, rotating at 4°, before centrifugation at 1,350*g* for 5 min at 4 °C. The pellet was lysed in buffer 2 (10 mM Tris pH 8, 200 mM NaCl, 1 mM EDTA and 0.5 mM EGTA), incubated for 10 min at 4 °C and once again centrifugated at 1,350*g* for 5 min. Then, the pellet was lysed in buffer 3 (10 mM Tris pH 8, 100 mM NaCl, 1 mM EDTA, 0.5 mM EGTA, 0.1% sodium deoxycholate and 0.5% *N*-lauroylsarcosine), incubated for 20 min on ice and sonicated in a Bioruptor sonicator (Diagenode) until the obtention of DNA fragments of sizes ranging 400–600 bp. Chromatin was quantified, and approximately 10–25 µg of chromatin were used for immunoprecipitation in a total of 500 µl of buffer 3 containing the antibodies indicated in Supplementary Table 1. After overnight incubation, 100 µl of magnetic protein G beads (10004D, Life Technologies) were added to each immunoprecipitation. After 2–3 h of incubation, the immunocomplexes were washed five times with RIPA wash buffer (50 mM HEPES-KOH pH 7.5, 500 mM LiCl, 1 mM EDTA, 1% NP-40 and 0.7% sodium deoxycholate) and once with TE-NaCl buffer (50 mM Tris pH 8, 10 mM EDTA and 50 mM NaCl). To recover the DNA, the immunocomplexes were eluted in elution buffer (50 mM Tris pH 8, 10 mM EDTA and 1% SDS) at 65 °C for 15 min with vortexing every 5 min. The bead eluate was decrosslinked overnight at 65 °C. After RNAse A treatment for 30 min (FEREN0531, Thermo Fisher Scientific) and proteinase K treatment (EO0492, Thermo Fisher) for 2 h, the DNA was purified using a Qiagen kit (28106, Qiagen).

To prepare ChIP–seq libraries for sequencing, we utilized the NEBNext Ultra II DNA kit (E7645S, NEB), and Agencourt AMPure XP beads (A63881, Beckman Coulter) were used for the cleanings. We started from approximately 20 to 50 ng of ChIP or input DNA and followed the manufacturer’s instructions. Paired-end sequencing (150 cycles) was performed in a Novaseq X Plus sequencer (Illumina) including 1% of PhiX.

### Bulk RNA-seq and library preparation

RNA was extracted using TRIzol from nontarg-CARGO and LTR5Hs-CARGO hnPSCs treated with cumate during 4 days in the absence of doxycycline, from ZNF729–FH hnPSCs treated with dTAG^v^-1 for 3 h and 24 h or from blastoids and dark spheres. mRNA was purified using poly-T oligo-attached magnetic beads. After fragmentation, the first-strand cDNA was synthesized using random hexamer primers followed by the second-strand cDNA synthesis. Libraries were prepared by end repair, A-tailing, adapter ligation, size selection, amplification and purification, and they were checked with Qubit and qPCR for quantification and Bioanalyzer for size-distribution detection. Quantified libraries will be pooled and sequenced on a Novaseq 6000 Illumina sequencer.

### Blastoid immunostainings

Immunostaining of blastoids was performed ‘in well’. Media from Aggrewell were carefully aspirated (more than 90%). For fixation, 1 ml of 4% paraformaldehyde was added to the well and incubated at room temperature for 15 min. The paraformaldehyde was carefully aspirated and substituted for a rinse buffer composed of PBS with 3 mg ml^−1^ polyvinylpyrrolidone. After one rinse, blastoids were permeabilized in PBS–polyvinylpyrrolidone containing 0.25% of Triton X-100 for 30 min. The permeabilization solution was aspirated substituted with blocking buffer (0.1% BSA (A9418, Sigma-Aldrich), 0.01% Tween 20 (P1379, Sigma-Aldrich) and 2% donkey serum (017-000-121, Jackson Immunoresearch)), which was dispensed in the well with a 5-ml serological pipette to subsequently collect all the blastoids from the well and deposit them into a well of a six-well plate containing more blocking solution. Blocking took place for at least 3 h at 4 °C. Blastoids were picked using standard mouth pipetting or 20-µl pipette tips and moved to primary antibodies (Supplementary Table 1) diluted in blocking solution in Nunc MicroWell MiniTrays (12-565-154, Fisher Scientific) at 4 °C overnight. Blastoids were washed three times with blocking buffer and stained with Alexa Fluor secondary antibodies for 3 h, washed three times and imaged in blocking buffer using an 18-well microslide (81826, Ibidi) in an Inverted Zeiss LSM 780 confocal microscope.

### PIP-seq

PIP-seq^70^ is an alternative to microfluidics-based scRNA-seq methods that captures cells via vortex and can be performed from beginning to library preparation at the experimenter’s bench. Blastoids from two wells of an Aggrewell plate per condition were collected and centrifuged in a 15-ml tube for 2 min at 250*g*, then the supernatant was aspirated. The blastoids pellet was resuspended in collagenase IV (07909, StemCell Technologies) and incubated at 37 °C with mild agitation for 40 min. Blastoids were centrifuged again in N2B27 medium and the pellet was resuspended in 0.5% trypsin-EDTA (15-400-054, Fisher Scientific) and incubated for 10 min at 37 °C. Two further washes were performed with N2B27, and the dissociated cells were passed through a 40-µm Flowmi cell strainer. Experiment only continued when viability was larger than 80%. Cells (40,000 or less) were counted, captured and used for completing the PIP-seq T20 3′ Single Cell RNA Kit protocol (FBS-SCR-T20-4-V4.05, Fluent Biosciences) without changes and using 12 cycles of cDNA amplification. Libraries were prepared with the reagents in the kit and were sequenced in an Illumina Novaseq X instrument.

### Western blot

After SDS–PAGE electrophoresis, protein transfer was carried out on a multilayered cassette including a nitrocellulose membrane. The transfer buffer was composed of 25 mM Tris-HCl, 192 mM glycine, 0.05% SDS and 10% methanol. The power source was set to 125 V for 90 min. The nitrocellulose membrane was blocked with 5% milk for 1 h and incubated with a HA tag antibody (Supplementary Table 1) overnight to detect ZNF729–HA or ZNF729–FH. For gel source data, see Supplementary Fig. 1.

### Image obtention and quantification

All bright-field images were taken using the EVOS FL Imaging System. The fluorescent immunostainings were imaged using an Inverted Zeiss LSM 780 confocal microscope. To obtain blastoids inner cell mass (ICM):trophectoderm (TE) ratios, we used the Fiji software^71^. We calculated the diameter of the blastoids cavity and the ICM size by measuring the distance from the point of contact with the trophectoderm to the end of the ICM. To count number of cells expressing specific lineage markers, we used a combination of software and manual counting. KLF17, GATA4 and cleaved CASP3-positive cells were counted with Fiji’s cell counter in each stack. GATA3-positive cells were counted using the 3D Object Counter plug-in from Fiji, carefully curating the assigned positive cells with the human eye and correcting when necessary (for example, fluorescent artefacts that are not cells).

### Quantification of blastoid formation efficiency

For determining blastoid efficiencies, end-point (96 h) blastoids were moved to a 15-ml conical tube and the total volume was measured. Next, two technical duplicates of 50-µl aliquots were dispensed into a 96-well plate and the structures were evaluated and counted, ultimately extrapolating to the total conical tube volume and to the 1,200 microwells present in the Aggrewell. To consider a 3D structure as a blastoid, we followed previously established criteria^17^. In brief, its morphology should resemble stage B6 of human blastocyst, with an accumulation of cells surrounded by a monolayered cyst mimicking the ICM and the trophectoderm, respectively. The ICM of the blastoids is often outside the cyst, in such case, we still considered that structure a blastoid. Blastoids have an approximate total diameter between 150 µm and 250 µm, and in the case of LTR5Hs-CARGO blastoids, the cavity should be larger than 150 µm, with no upper limit. When tested by scRNA-seq or immunofluorescence, blastoids must express markers consistent with the lineages of blastocyst. Dark spheres are structures that appear darker in bright field and are not cavitated. We note that the efficiencies calculated may be an underestimation, as some aggregates or blastoids are accidentally aspirated during the medium changes.

### Recording of blastoid formation video

The blastoid formation protocol was performed as indicated above, with changes. Instead of using Aggrewells (which have an opaque bottom), we utilized Elplasia 24-well plates that allow imaging from below (4441, Corning). We note that the initial cell aggregation in these plates is not as robust, thus end-point blastoid formation is less efficient than in Aggrewells. Cell aggregates are monitored, and when there are early signs of cavitation (small ‘bubbles’ around the aggregates), the plate is moved to a Nikon Eclipse Ti-E microscope that is equipped with a system for CO_2_ and temperature control (OKOlab). Blastoids were imaged at 37 °C and 5% CO_2_ for 24 h.

### CUT&RUN analysis

Standard Illumina adapters were cut from the Illumina reads using Cutadapt^72^ and then aligned to a combined hg38 and *E. coli* genome version using Bowtie2 (ref. ^73^), with the -dovetail parameter on and the rest of parameters in its default behaviour. This means that, in case of multimapping, all the valid alignments are reported. PCR duplicates were removed from the analysis. Coverage bigwig files were generated with Deeptools^74^ bamCoverage and the -scaleFactor was set to the number obtained from the normalization of fragments mapped to the human genome (hg38) and the mapped fragments to the *E. coli* k12 MG1655 genome. Browser captures were obtained from IGV^75^.

### ChIP–seq analysis

Standard Illumina adapters were cut from the Illumina reads using Cutadapt^72^. Reads were aligned to the *Homo sapiens* (hg38) genome using Bowtie2 (ref. ^73^) in its default behaviour. PCR duplicates were removed from the analysis using Samtools^76^. Coverage bigwig files were generated with Deeptools^74^ bamCoverage. Browser captures were obtained from IGV^75^.

Peaks were called using MACS3 (ref. ^77^). Identification of ZNF729–FH-bound repetitive DNA was performed by intersecting ZNF729–FH peaks with RepeatMasker (RRID:SCR_012954)^78^ using Bedtools^79^ intersect with -f 0.3. To be considered a peak at the promoter, ZNF729–FH or TRIM28 must bind to −1 kb or +200 bp around the transcription start site. Motif discovery analysis at the non-repetitive ZNF729 peaks was performed using the top 3,000 ZNF729-bound peaks using SeqPos^58^. Full SeqPos output can be found in Supplementary Table 7.

### RNA-seq and Gene Ontology analysis

Illumina adapters were trimmed from reads using Skewer^80^. Transcript alignment and quantification were performed using Salmon^81^ against the human genome assembly version Gencode (v47)^82^. For differential gene expression analysis, we used DESeq2 (ref. ^83^) after excluding transcripts with less than 10 reads across the tested samples. DESeq2 compared the effect of nontarg-CARGO and LTR5Hs-CARGO in hnPSCs, the differences between blastoids and dark spheres, or the gene expression changes upon dTAG^v^-1 addition to the ZNF729–FH hnPSCs. Biological replicates were used as covariates. Analysis of chimpanzee naive PSCs bulk RNA-seq^39^ was performed in the same manner but using the *Pan troglodytes* panTro6 Clint_PTRv2 genome assembly. The rhesus genome Mmul_10 (RheMac10) genome reference lacks a *ZNF729* transcript model. To assess the presence and expression of *ZNF729* in the rhesus macaques naive state, we performed unguided transcriptome assembly from rhesus monkey (*Macaca mulatta*) naive PSCs bulk RNA-seq data^40^ using the Trinity pipeline^84^. We constructed a blast database from the Trinity output and searched for the human *ZNF729* nucleotide sequence using blastn and tblastx algorithms^85^. The highest matches were searched against non-redundant NCBI database with blastn and blastx algorithms. None of the sequences scored *ZNF729* in reciprocal blast as a top match. When a sequence had a blast matching to *ZNF729*, such matches were below 60% identity. Differentially regulated genes (FDR 5%) were used for human Gene Ontology analysis using Gorilla^86^ using as a background the list of genes expressed in hnPSCs, blastoids and dark spheres.

### TEtranscripts

The software ‘TEtranscripts’^87^ was used to find differentially regulated transposable elements in the ZNF729–FH dTAG^v^-1 bulk RNA-seq experiments. To this end, the RNA-seq reads were aligned using HISAT2 (ref. ^88^), and following the tool’s manual, we allowed 100 alignments per read (-k 100) to optimize transposable element quantification and differential analysis. This analysis searches for differences at the level of transposable element families, so we cannot exclude the possibility that within a family, specific individual insertions could still be affected by ZNF729 depletion.

### PIP-seq and pseudobulk differential gene expression analysis

For each sample and replicate, reads obtained from the Novaseq X were analysed with Fluent Bio’s proprietary software Pipseeker with default parameters and aligning against the GRCh38 transcriptome index (Gencode v40 2022.04, Ensembl 106). A background removal step was performed in all samples using CellBender^89^ with parameters --fpr 0.01 and --epochs 150. Full count matrices with background RNA removed were further analysed using Seurat^90^. Cells with more than 10% of mitochondrial counts were eliminated and the number of genes detected was also used for filtering the data (see Supplementary Table 3 for specific parameters applied for each sample). Each object was normalized using Seurat’s LogNormalize method and transformed using the ScaleData function removing the unwanted variation originated from mitochondrial contamination or the cell-cycle stage. Upon examining elbow plots, 20 principal components were considered significant for unsupervised clustering with the FindNeighbors and FindClusters Seurat functions. Cluster identities were assigned based on the genes specifically marking each cluster according to Seurat’s FindMarkers function and comparing them with lineage markers uncovered in human pre-implantation datasets^18,27^. Seurat objects belonging to the nontarg-CARGO and the LTR5Hs-CARGO blastoids were merged and the LTR5Hs-CARGO object was downsampled to the same number of cells as the nontarg-CARGO object for comparison purposes. Multiple iterations of downsampling were performed with comparable results. We subset cells belonging to the epiblast or the neo-epiblast into a single group and performed differential gene expression analysis using DESeq2 on the sample-level aggregated counts (pseudobulk). Specifically, DESeq2 tested the effect of the repression of LTR5Hs elements (nontarg-CARGO versus LTR5Hs-CARGO) using the PIP-seq replicate as a covariate. Only genes with FDR of 5% and fold changes < −1 or fold changes > 1 were considered statistically significant. Genes related to the trophectoderm or placental development in Extended Data Fig. 5g are established markers or were obtained from Petropoulos et al.^18^ and other literature searches. Regarding the number of cells belonging to the different clusters, we do note that scRNA-seq may not be accurate for trophectoderm cell counting, as we have observed an accelerated lysis of trophectoderm cells upon blastoid dissociation before cell capture. In agreement with such possibility, the overall proportion of trophectoderm cells was systematically lower in our scRNA-seq analyses than in immunostainings, irrespective of the LTR5Hs activity status.

### Projection of transcriptomes into human embryo datasets

To identify the human embryo counterparts of the transcriptomes of cells dissociated from nontarg-CARGO or LTR5Hs-CARGO blastoids, we projected such transcriptomes into a collection of human embryo reference datasets^18,27,91–94^. Counts from each gene in each cell (slot ‘counts’ in Seurat) were extracted and uploaded to a human embryogenesis online prediction tool^51^ (https://petropoulos-lanner-labs.clintec.ki.se/). The identified annotations and UMAP values were used in our plots and conclusions.

### scRNA-seq data analysis including transposons

Raw data from Kagawa et al.^17^ was downloaded from the Gene Expression Omnibus database entry GSE177689. Smart-seq2 PCR adapters were trimmed using Skewer^80^ and the resulting reads were aligned using HISAT2 (ref. ^88^) with the parameters --dta --no-mixed --no-discordant -k 100, to allow enough multimappers for transposon analysis. The resulting bam files were processed with the scTE^95^ software for transposon family identification and quantification. The resulting matrix was subset to contain only cells dissociated from the 96-h blastoids and it was analysed using Seurat, filtering cells with more than 25% of mitochondrial counts, less than 2,000 or more than 16,000 genes. Downstream unsupervised clustering was performed using 20 principal components. For comparing blastoids from Kagawa et al. and this article, we integrated a nontarg-CARGO scRNA-seq object with the data from Kagawa et al. after removing the transposons using default Seurat data integration functions.

### Human–mouse and human–marmoset comparisons

LTR5Hs-regulated genes in the blastoids epiblast located within 250 kb of an LTR5Hs element were used as LTR5Hs target genes. To obtain genes expressed in the mouse and the marmoset epiblast, we used a previously published table containing expression levels and orthology analysis of human, mouse and marmoset genes. Specifically, we used the epiblast data (early ICM in the dataset)^34^. Only genes with an average expression of more than 2 fragments per kilobase million were considered expressed, with a caveat that this analysis may pass over more subtle, quantitative differences in expression between species, which can nonetheless be functionally important. This cut-off was validated by visual inspection of scRNA-seq datasets from mouse embryos^96^. Genes were assigned to evolutionary branches using data from the Gentree database^38^.

### *ZNF729* locus conservation

Figure 3e is a cartoon depiction of Zoonomia project’s Cactus genomic alignments^53,54^ in the UCSC genome browser^97^.

### Reporting summary

Further information on research design is available in the Nature Portfolio Reporting Summary linked to this article.

## Online content

Any methods, additional references, Nature Portfolio reporting summaries, source data, extended data, supplementary information, acknowledgements, peer review information; details of author contributions and competing interests; and statements of data and code availability are available at 10.1038/s41586-025-09571-1.

## Supplementary information

Supplementary Figure 1 **a**. Uncropped blots corresponding to Fig. 4b. Left membrane displays a blot using anti-HA tag polyclonal antibody. Right membrane displays a blot using anti-α-ACTIN. **b**. Uncropped blots corresponding to Extended Data Fig. 7I. Left membrane displays a blot using anti-HA tag monoclonal antibody. Right membrane a blot using anti-β-ACTIN.

Reporting Summary

Supplementary Tables Supplementary Tables 1–8.

Supplementary Video 1 **Example of blastoid formation**. Blastoid formation in Elplasia plates. Images recorded from ~80-103 h after plating wild type hnPSCs in N2B27 supplemented with Y-27632.

## Source data

Source Data Fig. 1

Source Data Fig. 2

Source Data Fig. 3

Source Data Extended Data Fig. 1

Source Data Extended Data Fig. 2

Source Data Extended Data Fig. 3

Source Data Extended Data Fig. 5

Source Data Extended Data Fig. 6

Source Data Extended Data Fig. 7

## Data Availability

Datasets generated in this article have been deposited in the Gene Expression Omnibus repository: GSE262191 for CUT&RUN, GSE296554 for bulk RNA-seq, GSE262329 for PIP-seq and GSE296555 for ChIP–seq. Source data are provided with this paper.

## Code availability

No custom code was used; only previously published pipelines were implemented.
