# Supplementary material for: A human-specific regulatory mechanism revealed in a pre-implantation model
Source: Nature. 2025 Oct 1;647(8088):238–47. doi: 10.1038/s41586-025-09571-1 (PMC12589118; doi:10.1038/s41586-025-09571-1)
Supplement: Supplementary file 1 — a. Uncropped blots corresponding to Fig. 4b. Left membrane displays a blot using anti-HA tag polyclonal antibody. Right membrane displays a blot using anti-α-ACTIN. b. Uncropped blots corresponding to Extended Data Fig. 7I. Left membrane displays a blot using anti-HA tag monoclonal antibody. Right membrane a blot using anti-β-ACTIN. [file 41586_2025_9571_MOESM1_ESM.pdf]

nature portfolio

https://doi.org/10.1038/s41586-025-09571-1

Supplementary information

A human-specific regulatory mechanism revealed in a pre-implantation model

In the format provided by the authors and unedited

A human-specific regulatory mechanism revealed in a preimplantation model

Raquel Fueyo[1], Sicong Wang[2], Olivia J. Crocker[3], Tomek Swigut[1], Hiromitsu Nakauchi[2,5], Joanna Wysocka[1,2,4,5*]

[1] Department of Chemical and Systems Biology, Stanford University School of Medicine, Stanford, CA 94305, USA

[2] Institute of Stem Cell Biology and Regenerative Medicine, Stanford University School of Medicine, Stanford, CA 94305, USA

[3] Department of Genetics, Stanford University School of Medicine, Stanford, CA 94305, USA

[4] Department of Developmental Biology, Stanford University School of Medicine, Stanford, CA 94305, USA

[4] Howard Hughes Medical Institute, Stanford University School of Medicine, Stanford, CA 94305, USA

[5] Stem Cell Therapy Laboratory, Advanced Research Institute, Tokyo Medical and Dental University,

1-5-45 Yushima, Bunkyo-ku, Tokyo 113-8510, Japan

* Lead contact, corresponding author

Contact info: wysocka@stanford.edu

Table of contents:

Supplementary Figure 1. Uncropped western blots

A

Corresponding to Figure 4B, top

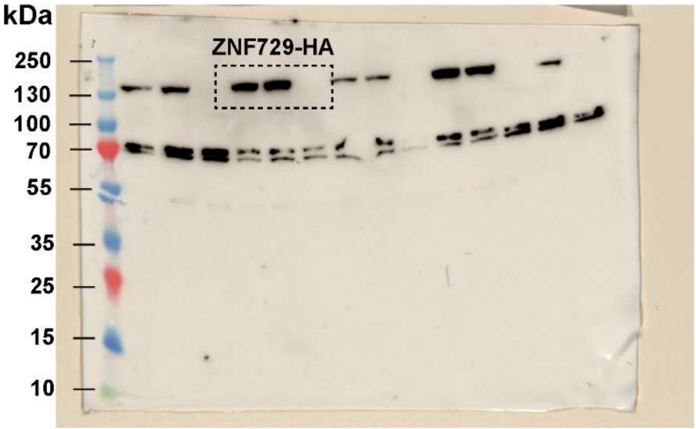

Corresponding to Figure 4B, bottom

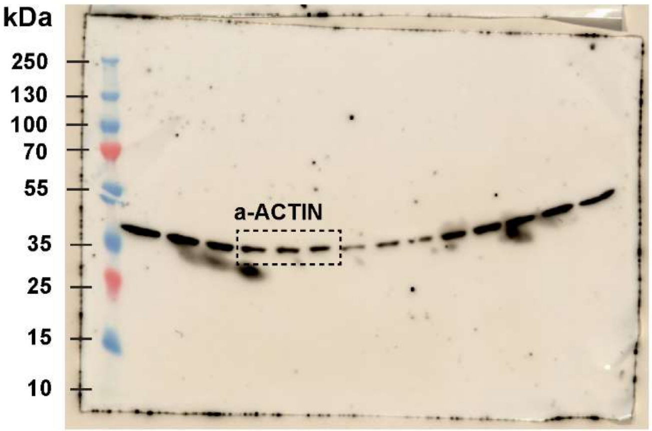

B

Corresponding to Extended Data Figure 7I, top

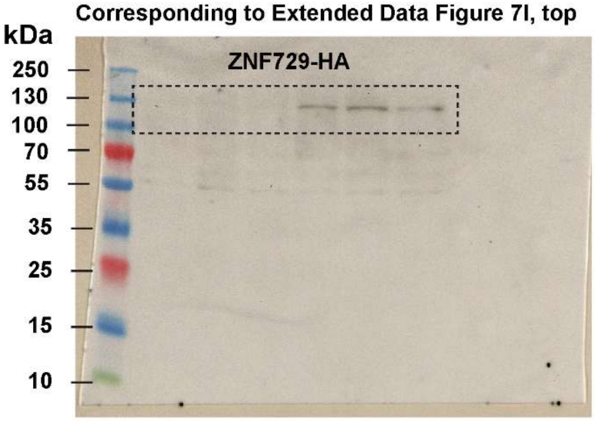

Corresponding to Extended Data Figure 7I, bottom

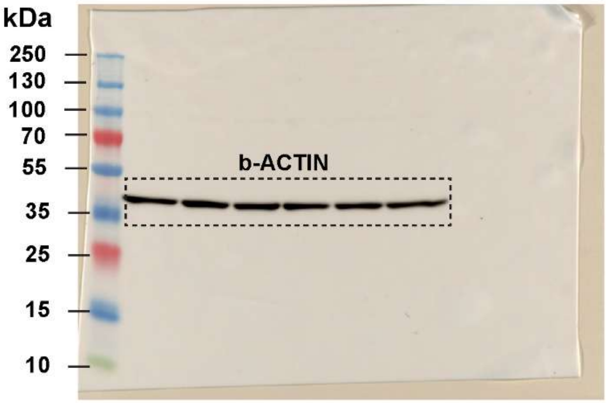

Supplementary Figure 1. Uncropped western blots
