# Supplementary material for: A human-specific regulatory mechanism revealed in a pre-implantation model
Source: Nature. 2025 Oct 1;647(8088):238–47. doi: 10.1038/s41586-025-09571-1 (PMC12589118; doi:10.1038/s41586-025-09571-1)
Supplement: Supplementary file 2 — Reporting Summary [file 41586_2025_9571_MOESM2_ESM.pdf]

nature portfolio

Corresponding author(s): Joanna Wysocka

Last updated by author(s): Jul 24, 2025

Reporting Summary

Nature Portfolio wishes to improve the reproducibility of the work that we publish. This form provides structure for consistency and transparency in reporting. For further information on Nature Portfolio policies, see our Editorial Policies and the Editorial Policy Checklist.

Statistics

For all statistical analyses, confirm that the following items are present in the figure legend, table legend, main text, or Methods section.

n/a	Confirmed	
☐	☒	The exact sample size (*n*) for each experimental group/condition, given as a discrete number and unit of measurement
☐	☒	A statement on whether measurements were taken from distinct samples or whether the same sample was measured repeatedly
☐	☒	The statistical test(s) used AND whether they are one- or two-sided<br>*Only common tests should be described solely by name; describe more complex techniques in the Methods section.*
☐	☒	A description of all covariates tested
☒	☐	A description of any assumptions or corrections, such as tests of normality and adjustment for multiple comparisons
☐	☒	A full description of the statistical parameters including central tendency (e.g. means) or other basic estimates (e.g. regression coefficient) AND variation (e.g. standard deviation) or associated estimates of uncertainty (e.g. confidence intervals)
☐	☒	For null hypothesis testing, the test statistic (e.g. *F*, *t*, *r*) with confidence intervals, effect sizes, degrees of freedom and *P* value noted<br>*Give P values as exact values whenever suitable.*
☒	☐	For Bayesian analysis, information on the choice of priors and Markov chain Monte Carlo settings
☒	☐	For hierarchical and complex designs, identification of the appropriate level for tests and full reporting of outcomes
☐	☒	Estimates of effect sizes (e.g. Cohen's *d*, Pearson's *r*), indicating how they were calculated

Our web collection on statistics for biologists contains articles on many of the points above.

Software and code

Policy information about availability of computer code

Data collection	Flow cytometry data were collected on a SONY MA900. qPCR data on a LightCycler 480 II instrument. CUT&RUN and bulk RNA-seq data on a Novaseq 6000 Illumina sequencer. ChIP-seq and single-cell RNA-seq data on a Novaseq X Plus Illumina sequencer. Immunofluorescence images were taken on an Inverted Zeiss LSM 780 confocal microscope. Bright field images on an EVOS FL Imaging System. Blastoids video was taken on a Nikon Eclipse Ti-E. Western blots were revealed on an Amersham ImageQuant 800.
Data analysis	Flow cytometry was analyzed with FlowJo v.10.10.0. Fiji/ImageJ v1.54 was used for microscopy image analysis and measurements. In sequencing data analyses, we used Cutadapt v4.6 to trim Illumina adaptors (in RNA-seq adaptor trimming was performed using Skewer v0.2.2). Bowtie v2.5.1 and Hisat2 v2.2.1 were used to map sequencing data. Transcript alignment was performed with Salmon v1.4.0. Samtools v1.10 to remove PCR duplicates. Peaks were called with MACS3 v3.0.1 Deeptools v3.5.3 to generate bigwigs. Browser captures were obtained in IGV v2.16.0. Differential gene expression analysis was performed in RStudio Build 421 using DESeq2 v1.42.0. ZNF729 transcript in Rhesus was searched using Blast v2.15.0 and Trinity v2.15.2. TEtranscripts v2.2.3. Count matrices from PIP-seq data were obtained with Pipseeker v3.0.5 and CellBender v0.3.0. Downstream analyses were performed in RStudio Build 421 using Seurat v4.4.0

For manuscripts utilizing custom algorithms or software that are central to the research but not yet described in published literature, software must be made available to editors and reviewers. We strongly encourage code deposition in a community repository (e.g. GitHub). See the Nature Portfolio guidelines for submitting code & software for further information.

Data

Policy information about availability of data

All manuscripts must include a data availability statement. This statement should provide the following information, where applicable:

- Accession codes, unique identifiers, or web links for publicly available datasets
- A description of any restrictions on data availability
- For clinical datasets or third party data, please ensure that the statement adheres to our policy

Datasets generated in this manuscript can be found at Gene Expression Omnibus (GEO). Accessions: GSE262191, GSE296554 , GSE296555, and GSE262329. Datasets were aligned to the human genome assembly hg38. Publicly available datasets analyzed: Petropoulos et al. Human embryo scRNA-seq (ArrayExpress: E-MTAB-3929), Kagawa et al. Blastoids scRNA-seq (GEO GSE177689). Human naïve pluripotent stem cells bulk RNA-seq: naïve_H9 (GEO GSE131747 and GSE144994), HNES1 and cRH9 GSE123055. Chimpanzee naïve pluripotent stem cells (GEO: GSE264735, genome assembly panTro6 Clint_PTRv2). Macaque naïve pluripotent stem cells (GEO: GSE61420, genome assembly Mmul_10 (RheMac10)). Reference human embryo datasets for projections (ArrayExpress E-MTAB-3929, GEO GSE36552, GEO GSE136447, ArrayExpress E-MTAB-9388, ENA PRJEB30442, GEO GSE171820). KRAB zinc finger protein compendium KRABopedia: https://krabopedia.org/. RepeatMasker (RRID:SCR_012954). Isoform resolved human embryo transcriptome (https://denis-torre.github.io/embryo-transcriptome/#genome-browser).

Research involving human participants, their data, or biological material

Policy information about studies with human participants or human data. See also policy information about sex, gender (identity/presentation), and sexual orientation and race, ethnicity and racism.

Reporting on sex and gender	N/A
Reporting on race, ethnicity, or other socially relevant groupings	N/A
Population characteristics	N/A
Recruitment	N/A
Ethics oversight	This work was performed following the 2021 ISSCR Guidelines50. The use of blood-derived induced naive pluripotent stem cells for the experiments described in this manuscript was approved by the Stanford Stem Cell Research Oversight committee (SCRO Protocol number 900).

Note that full information on the approval of the study protocol must also be provided in the manuscript.

Field-specific reporting

Please select the one below that is the best fit for your research. If you are not sure, read the appropriate sections before making your selection.

☒ Life sciences ☐ Behavioural & social sciences ☐ Ecological, evolutionary & environmental sciences

For a reference copy of the document with all sections, see nature.com/documents/nr-reporting-summary-flat.pdf

Life sciences study design

All studies must disclose on these points even when the disclosure is negative.

Sample size	No statistical methods were used to predetermine sample size. The sample sizes presented in this manuscript were selected aiming to capture the variability of the experiments based on our laboratory experience. For new approaches, such as measuring blastoid formation potential vs. LTR5Hs activity, for which experimental variability was a concern, we derived tenths of clones instead of the usual 2 or 3 clones per condition, and performed more than two replicates with each clone. For RNA-seq (bulk or single-cell) we collected a sample size sufficient to perform differential gene expression analysis, n≥2 biological replicates from each sample. For imaging experiments we followed common practices in the developmental biology field (i.e. staining multiple structures from different biological replicates) to capture variability across cell passages and across experiments. For ChIP-seq experiments we performed two independent biological replicates. We deemed this sufficient because the replicates correlated and we had dTAG controls for the specificity.
Data exclusions	In the PIP-seq data, low quality cells were discarded and the specific cut-offs have been included in the Supplementary Table 3.
Replication	All the experiments were biologically replicated and when applicable, at least two clones were used per condition.
Randomization	Blastoids/dark spheres picked for stainings were randomly selected from the pool of blastoids in the plate.
Blinding	We did not apply any blinding approach to our experiments because all samples were treated with cumate. In the case of dTAG experiments blinding is not possible because cells start growing slower.

Reporting for specific materials, systems and methods

We require information from authors about some types of materials, experimental systems and methods used in many studies. Here, indicate whether each material, system or method listed is relevant to your study. If you are not sure if a list item applies to your research, read the appropriate section before selecting a response.

Materials & experimental systems

n/a	Involved in the study
☐	☒ Antibodies
☐	☒ Eukaryotic cell lines
☒	☐ Palaeontology and archaeology
☒	☐ Animals and other organisms
☒	☐ Clinical data
☒	☐ Dual use research of concern
☒	☐ Plants

Methods

n/a	Involved in the study
☐	☒ ChIP-seq
☐	☒ Flow cytometry
☒	☐ MRI-based neuroimaging

Antibodies

Antibodies used

Primary antibodies:
H3K9me3, CUT&RUN, Abcam ab8898, lot 1063770-1, 0.5 ug
HERVK-envelope, Immunostaining, Austral Biological 1811-5, clone 6B7/14, lot MA170221HK, 1:1000
KLF17, Immunostaining, Sigma HPA024629, lot 000044816, 1:200
GATA3, Immunostaining, R&D Systems AF2605, lot UZQ0223041, 1:200
GATA4, Immunostaining, EBioEvan 14-9980-82, clone Ebioevan, lot 2448555, 1:100
SUSD2, Immunostaining, R&D Systems MAB90562, clone 1279A, lot CKHB0121031, 1:100
Cleaved-CASP3, Immunostaining, Cell Signaling Technology 9661, lot unknown, 1:200
NANOG, Immunostaining, Abcam ab109250, clone [EPR2027(2)], lot 1001613-39, 1:100
SOX17, Immunostaining, R&D Systems AF1924, lot KGA1021031, 1:200
IFI16, Immunostaining, Cell Signaling Technology 14970, clone D8B5T, lot 1, 1:100
TROP2 (TACSTD2), Flow cytometry, BD Biosciences 563243, clone 16246, lot 2320729, 1:1000
ANPEP, Flow cytometry, BioLegend 301716, clone WM15, lot B395908, 1:200
HA-tag monoclonal, Western Blot, Abcam ab18181, clone [HA.C5], 1:1000
α-ACTIN, Western Blot, Sigma CBL171-I, clone ASM-1, lot unknown, 0.5ug/ml
β-ACTIN, Western Blot, Abcam ab49900, clone AC15, lot 1055692-1 , 1:5000
HA-tag polyclonal ChIP-seq / Western Blot, Abcam 9110, lot GR146572, 5 ug / 1:2000
TRIM28, ChIP-seq, GeneTex GTX102226, clone NC32, lot unknown, 5 ug
H3K4me3, ChIP-seq, Active motif 39159, lot 22118006, 5 ug
H3K27ac, ChIP-seq, Active Motif 39133, lot 31521015, 5 ug
Secondary antibodies:
Alexa Fluor Donkey anti-mouse Plus 405, Immunostaining, invitrogen A48257, lot XB342724, 1:500
Alexa Fluor Donkey anti-mouse 488, Immunostaining, invitrogen A21202, lot 1796367, 1:500
Alexa Fluor Donkey anti-mouse 647, Immunostaining, Life technologies A31571, lot 1757130, 1:500
Alexa Fluor Donkey anti-rabbit 405, Immunostaining, invitrogen A48258, lot XA344369, 1:500
Alexa Fluor Donkey anti-rabbit 647, Immunostaining, life technologies A31573, lot 1693297, 1:500
Alexa Fluor Donkey anti-rat 405, Immunostaining, invitrogen A48268, lot WJ337984, 1:500
Alexa Fluor Donkey anti-rat 647, Immunostaining , invitrogen A48272TR, lot XG354854, 1:500

Validation

All the antibodies have been validated by the vendor for human. Validation websites are indicated:
H3K9me3 https://www.abcam.com/en-us/products/primary-antibodies/histone-h3-tri-methyl-k9-antibody-chip-grade-ab8898#overlay=images
HERVK-envelope http://www.australbiologicals.com/index.php?what=catalog&id=338 & PMID:30070637
KLF17 https://www.sigmaaldrich.com/US/en/product/sigma/hpa024629
GATA3 https://www.rndsystems.com/products/human-gata-3-antibody_af2605
GATA4 https://www.thermofisher.com/antibody/product/Gata-4-Antibody-clone-eBioEvan-Monoclonal/14-9980-82
SUSD2 https://www.rndsystems.com/products/human-susd2-antibody-1279a_mab90562
Cleaved-CASP3 https://www.cellsignal.com/products/primary-antibodies/cleaved-caspase-3-asp175-antibody/9661/applications?index=1&application=IF-IC&type=pdp
NANOG https://www.abcam.com/en-us/products/primary-antibodies/nanog-antibody-epr20272-ab109250
SOX17 https://www.rndsystems.com/products/human-sox17-antibody_af1924
IFI16 https://www.cellsignal.com/products/primary-antibodies/ifi16-d8b5t-rabbit-mab/14970
TROP2 (TACSTD2) https://www.bdbiosciences.com/en-us/products/reagents/flow-cytometry-reagents/research-reagents/single-color-antibodies-ruo/bv421-mouse-anti-human-trop-2.563243?tab=product_details
ANPEP https://www.biolegend.com/ja-jp/products/brilliant-violet-421-anti-human-cd13-antibody-8895
HA-tag monoclonal https://www.abcam.com/en-us/products/primary-antibodies/ha-tag-antibody-hac5-ab18181
α-ACTIN https://www.sigmaaldrich.com/US/en/product/mm/cbl171i
β-ACTIN https://www.abcam.com/en-us/products/primary-antibodies/hrp-beta-actin-antibody-ac-15-loading-control-ab49900

HA-tag polyclonal https://www.abcam.com/en-us/products/primary-antibodies/ha-tag-antibody-chip-grade-ab9110#overlay=images
TRIM28 https://www.genetex.com/Product/Detail/KAP1-antibody-N3C2-Internal/GTX102226
H3K4me3 https://www.activemotif.com/catalog/details/39159/histone-h3-trimethyl-lys4-antibody-pab
H3K27ac https://www.activemotif.com/catalog/details/39133/histone-h3-acetyl-lys27-antibody-pab

Eukaryotic cell lines

Policy information about cell lines and Sex and Gender in Research

Cell line source(s)	Peripheral blood cells were donated from consenting volunteers to establish hnPSCs at the University of Tokyo. Official name: PB004 Human iPSC line. Cells were generated in this study: PMID: 26023098
Authentication	STR analysis
Mycoplasma contamination	Cells were routinely tested for mycoplasm. and tested negative
Commonly misidentified lines (See ICLAC register)	No common misidentified cell lines were used in this study.

Plants

Seed stocks	*Report on the source of all seed stocks or other plant material used. If applicable, state the seed stock centre and catalogue number. If plant specimens were collected from the field, describe the collection location, date and sampling procedures.*
Novel plant genotypes	*Describe the methods by which all novel plant genotypes were produced. This includes those generated by transgenic approaches, gene editing, chemical/radiation-based mutagenesis and hybridization. For transgenic lines, describe the transformation method, the number of independent lines analyzed and the generation upon which experiments were performed. For gene-edited lines, describe the editor used, the endogenous sequence targeted for editing, the targeting guide RNA sequence (if applicable) and how the editor was applied.*
Authentication	*Describe any authentication procedures for each seed stock used or novel genotype generated. Describe any experiments used to assess the effect of a mutation and, where applicable, how potential secondary effects (e.g. second site T-DNA insertions, mosiacism, off-target gene editing) were examined.*

ChIP-seq

Data deposition

☒ Confirm that both raw and final processed data have been deposited in a public database such as GEO.

☒ Confirm that you have deposited or provided access to graph files (e.g. BED files) for the called peaks.

Data access links
May remain private before publication.

GSE296555. Called peaks can be found in Supplementary Table 6, hg38.

Files in database submission

Raw data files
INPUT_DMSO_REP1_1.fq.gz INPUT_DMSO_REP1_2.fq.gz
ZNF729-HA_DMSO_REP1_1.fq.gz ZNF729-HA_DMSO_REP1_2.fq.gz
INPUT_dTAG_REP1_1.fq.gz INPUT_dTAG_REP1_2.fq.gz
ZNF729-HA_dTAG_REP1_1.fq.gz ZNF729-HA_dTAG_REP1_2.fq.gz
INPUT_DMSO_REP2_1.fq.gz INPUT_DMSO_REP2_2.fq.gz
ZNF729-HA_DMSO_REP2_1.fq.gz ZNF729-HA_DMSO_REP2_2.fq.gz
INPUT_dTAG_REP2_1.fq.gz INPUT_dTAG_REP2_2.fq.gz
ZNF729-HA_dTAG_REP2_1.fq.gz ZNF729-HA_dTAG_REP2_2.fq.gz
INPUT_DMSO_ZNF729FH_REP1_1.fq.gz INPUT_DMSO_ZNF729FH_REP1_2.fq.gz
TRIM28_DMSO_ZNF729FH_REP1_1.fq.gz TRIM28_DMSO_ZNF729FH_REP1_2.fq.gz
INPUT_dTAG_ZNF729FH_REP1_1.fq.gz INPUT_dTAG_ZNF729FH_REP1_2.fq.gz
TRIM28_dTAG_ZNF729FH_REP1_1.fq.gz TRIM28_dTAG_ZNF729FH_REP1_2.fq.gz
INPUT_DMSO_ZNF729FH_REP2_1.fq.gz INPUT_DMSO_ZNF729FH_REP2_2.fq.gz
TRIM28_DMSO_ZNF729FH_REP2_1.fq.gz TRIM28_DMSO_ZNF729FH_REP2_2.fq.gz
INPUT_dTAG_ZNF729FH_REP2_1.fq.gz INPUT_dTAG_ZNF729FH_REP2_2.fq.gz
TRIM28_dTAG_ZNF729FH_REP2_1.fq.gz TRIM28_dTAG_ZNF729FH_REP2_2.fq.gz
H3K4me3_REP1_1.fq.gz H3K4me3_REP1_2.fq.gz
H3K4me3_REP2_1.fq.gz H3K4me3_REP2_2.fq.gz
H3K27ac_REP1_1.fq.gz H3K27ac_REP1_2.fq.gz
H3K27ac_REP2_1.fq.gz H3K27ac_REP2_2.fq.gz
Processed data files
INPUT_DMSO_REP1.bw
ZNF729-HA_DMSO_REP1.bw
INPUT_dTAG_REP1.bw
ZNF729-HA_dTAG_REP1.bw
INPUT_DMSO_REP2.bw

	ZNF729-HA_DMSO_REP2.bw<br>INPUT_dTAG_REP2.bw<br>ZNF729-HA_dTAG_REP2.bw<br>INPUT_DMSO_ZNF729FH_REP1.bw<br>TRIM28_DMSO_ZNF729FH_REP1.bw<br>INPUT_dTAG_ZNF729FH_REP1.bw<br>TRIM28_dTAG_ZNF729FH_REP1.bw<br>INPUT_DMSO_ZNF729FH_REP2.bw<br>TRIM28_DMSO_ZNF729FH_REP2.bw<br>INPUT_dTAG_ZNF729FH_REP2.bw<br>TRIM28_dTAG_ZNF729FH_REP2.bw<br>H3K4me3_REP1.bw<br>H3K4me3_REP2.bw<br>H3K27ac_REP1.bw<br>H3K27ac_REP2.bw
Genome browser session (e.g. UCSC)	No longer applicable

Methodology

Replicates	Two biologically independent replicates per ChIP
Sequencing depth	20-40 M reads
Antibodies	HA tag: Abcam 9110, TRIM28 GeneTex GTX102226, H3K4me3 Active motif 39159 , H3K27ac Active Motif 39133
Peak calling parameters	MACS3 software, -q 0.05. Narrow peaks
Data quality	Data quality was assessed by Novogene and by ourselves
Software	Our quality control analysis was performed with FASTP

Flow Cytometry

Plots

Confirm that:

☒ The axis labels state the marker and fluorochrome used (e.g. CD4-FITC).

☒ The axis scales are clearly visible. Include numbers along axes only for bottom left plot of group (a 'group' is an analysis of identical markers).

☒ All plots are contour plots with outliers or pseudocolor plots.

☒ A numerical value for number of cells or percentage (with statistics) is provided.

Methodology

Sample preparation	After 3 days of trophectoderm or hypoblast differentiation cells were used for staining.<br>Cells were pelleted and resuspended in 100 uL of N2B27 supplemented with 10 uM Y-27632
Instrument	SONY MA900
Software	FlowJo v.10.10.0.
Cell population abundance	Percentage of differentiated cells was defined using the negative control.
Gating strategy	Gating exammple can be found in Extended Data Figure 4E and 4H. Debris and cellular aggregates were removed from analysis via FSC-A/SSC-A gating following by FSC-A/FSC-W and SSC-A/SSC-W gating.<br>Positive signal was defined as cells not overlapping with the negative control (unstained cells).

☒ Tick this box to confirm that a figure exemplifying the gating strategy is provided in the Supplementary Information.
