# Supplementary material for: A human-specific regulatory mechanism revealed in a pre-implantation model
Source: Nature. 2025 Oct 1;647(8088):238–47. doi: 10.1038/s41586-025-09571-1 (PMC12589118; doi:10.1038/s41586-025-09571-1)
Supplement: Supplementary file 3 — Supplementary Tables 1–8. [file 41586_2025_9571_MOESM3_ESM.zip › 2024-03-06271B-s3/SupplementaryTablelegends.docx]

**Supplementary Table 1.** **List of reagents utilized in this manuscript**

Primers, probes, gRNAs and antibodies with dilutions for each method.

**Supplementary Table 2.** **Gene ontology analysis**

Gene ontology categories obtained upon testing with GOrilla the differentially regulated genes between medium and high LTR5Hs repression clones and between blastoids and dark spheres.

**Supplementary Table 3.** **PIP-seq filtering parameters**

Parameters for filtering the count matrices of the PIP-seq sequencing data based on mitochondrial counts and number of genes.

**Supplementary Table 4.** **Differential gene expression analysis in the blastoids epiblast-like cells**

Differentially regulated genes between the LTR5Hs-CARGO and nontarg-CARGO blastoids epiblast.

**Supplementary Table 5.**  **Gene expression and conservation in humans, mice, and marmosets**

Summary of gene expression (FPKM) and conservation between LTR5Hs-regulated genes in the human blastoids epiblast, mice, and marmosets based on data in Boroviak et al.

**Supplementary Table 6.** **Coordinates of ChIP-seq peaks**

ChIP-seq peaks of ZNF729-HA in DMSO control conditions and upon 24 h of dTAG^v^-1 treatment in ZNF729-FH cells. ChIP-seq peaks of TRIM28 in DMSO control conditions and upon 24 h of dTAG^v^-1 treatment in ZNF729-FH cells.

**Supplementary Table 7.** **SeqPos output**

SeqPos output of motif analysis performed using the top 3,000 ZNF729-bound non-repetitive regions.

**Supplementary Table 8.** **Gene ontology analysis**

GOrilla gene ontology analysis of transcripts misregulated upon 3 h of dTAG^v^-1 treatment in ZNF729-FH hnPSCs.
